# Supplementary material for: Tunable phenotypic variability through an autoregulatory alternative sigma factor circuit
Source: Mol Syst Biol. 2021 Jul 19;17(7):e9832. doi: 10.15252/msb.20209832 (PMC8287880; doi:10.15252/msb.20209832)
Supplement: Supplementary file 1 — Appendix [file MSB-17-e9832-s006.pdf]

Tunable phenotypic variability through an autoregulatory alternative sigma factor circuit

Christian P. Schwall[1,†], Torkel E. Loman[1,†], Bruno M.C. Martins[1,2], Sandra Cortijo[1], Casandra Villava[1], Vassili Kusmartsev[1], Toby Livesey[1], Teresa Saez[1], James C. W. Locke[1,*]

[1]Sainsbury Laboratory, University of Cambridge, Bateman Street, Cambridge CB2 1LR, UK
[2]University of Warwick, School of Life Sciences, Coventry CV4 7AL, UK
[*] Corresponding author. Tel: +44 1223 76111. Email: james.locke@slcu.cam.ac.uk
[†] These authors contributed equally.

Appendix

Table of Contents

1. Appendix Figures 2
2. Appendix Tables 52

1. Appendix Figures

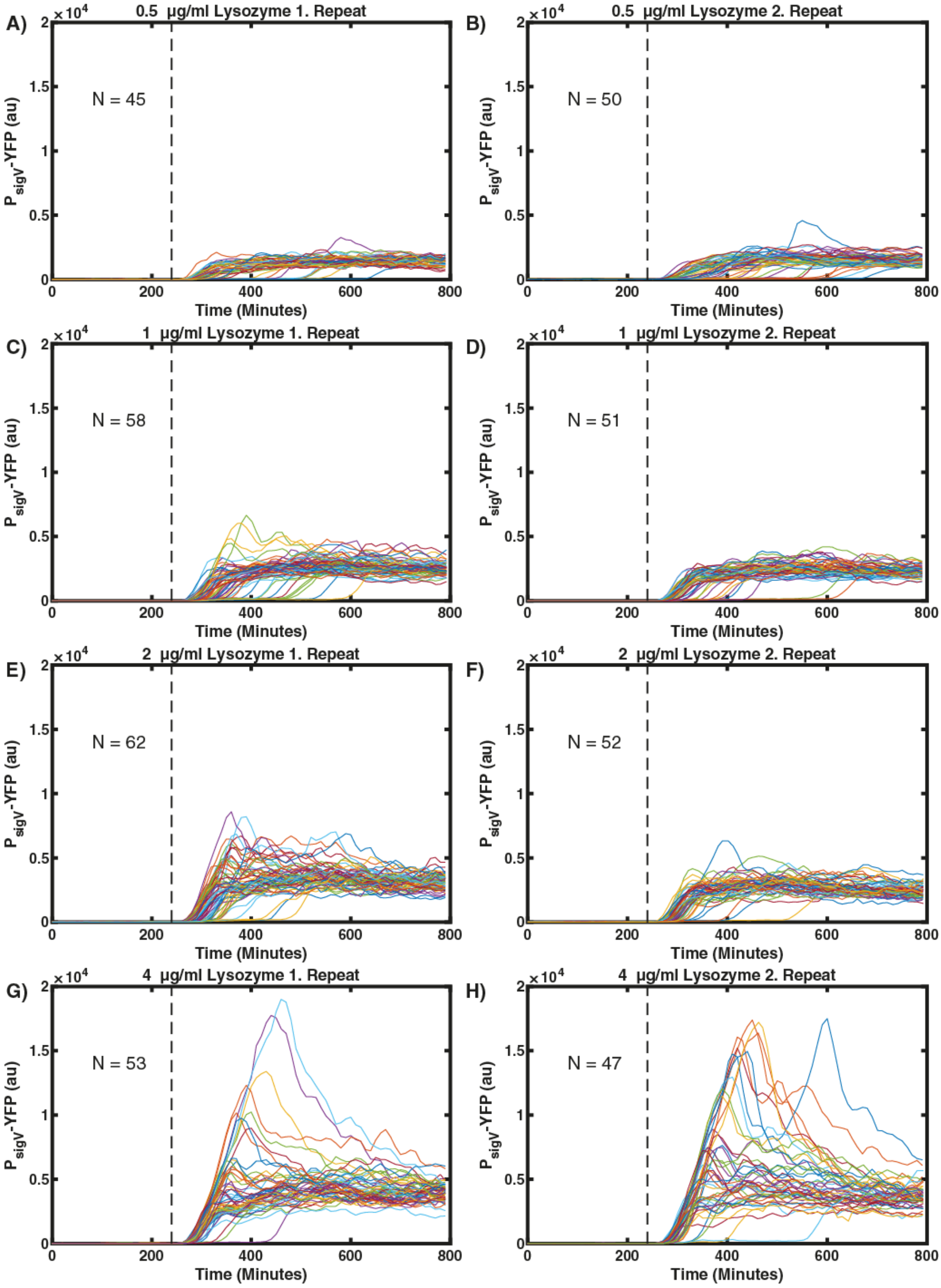

Appendix Figure S1. Expression dynamics of P_{sigV}-YFP reporter under lysozyme stress.

In each subpanel a line corresponds to a single-cell trace of one mother cell in the mother machine (N=~50). The stress was added after 240 min (dashed line).

(A&B) Single-cell traces in response to 0.5 µg/ml lysozyme. (C&D) Single-cell traces in response to 1 µg/ml lysozyme. (E&F) Single-cell traces in response to 2 µg/ml lysozyme. (G&H) Single-cell traces in response to 4 µg/ml lysozyme. The shown data corresponds to the data shown in Figure 1 and Figure EV1.

Data information: For more information on the number of repeats please see Appendix Table S5.

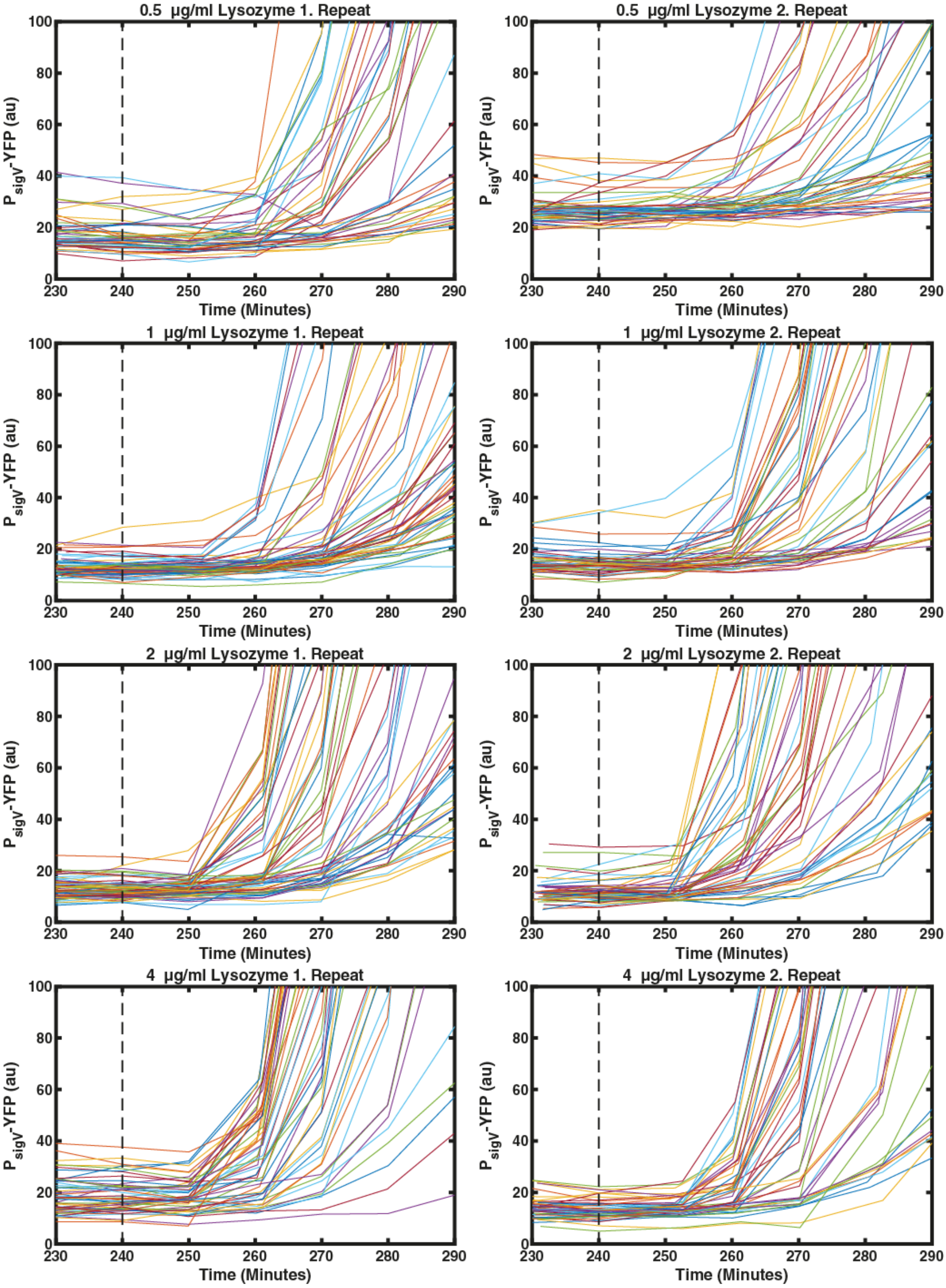

Appendix Figure S2. A fraction of cells respond rapidly to lysozyme.

The first cells begin to responds to the lysozyme stress with raised P_{sigV}-YFP levels within 2 frames (20 minutes). In each subpanel a line corresponds to a single-cell trace of one mother cell in the mother machine (N=~50). The stress was added after 240 min (dashed line). (A&B) Single-cell traces in response to 0.5 µg/ml lysozyme. (C&D) Single-cell traces in response to 1 µg/ml lysozyme. (E&F) Single-cell traces in response to 2 µg/ml lysozyme. (G&H) Single-cell traces in response to 4 µg/ml lysozyme. The shown data is the same as in Figure S1.

Data information: For more information on the number of repeats please see Appendix Table S5.

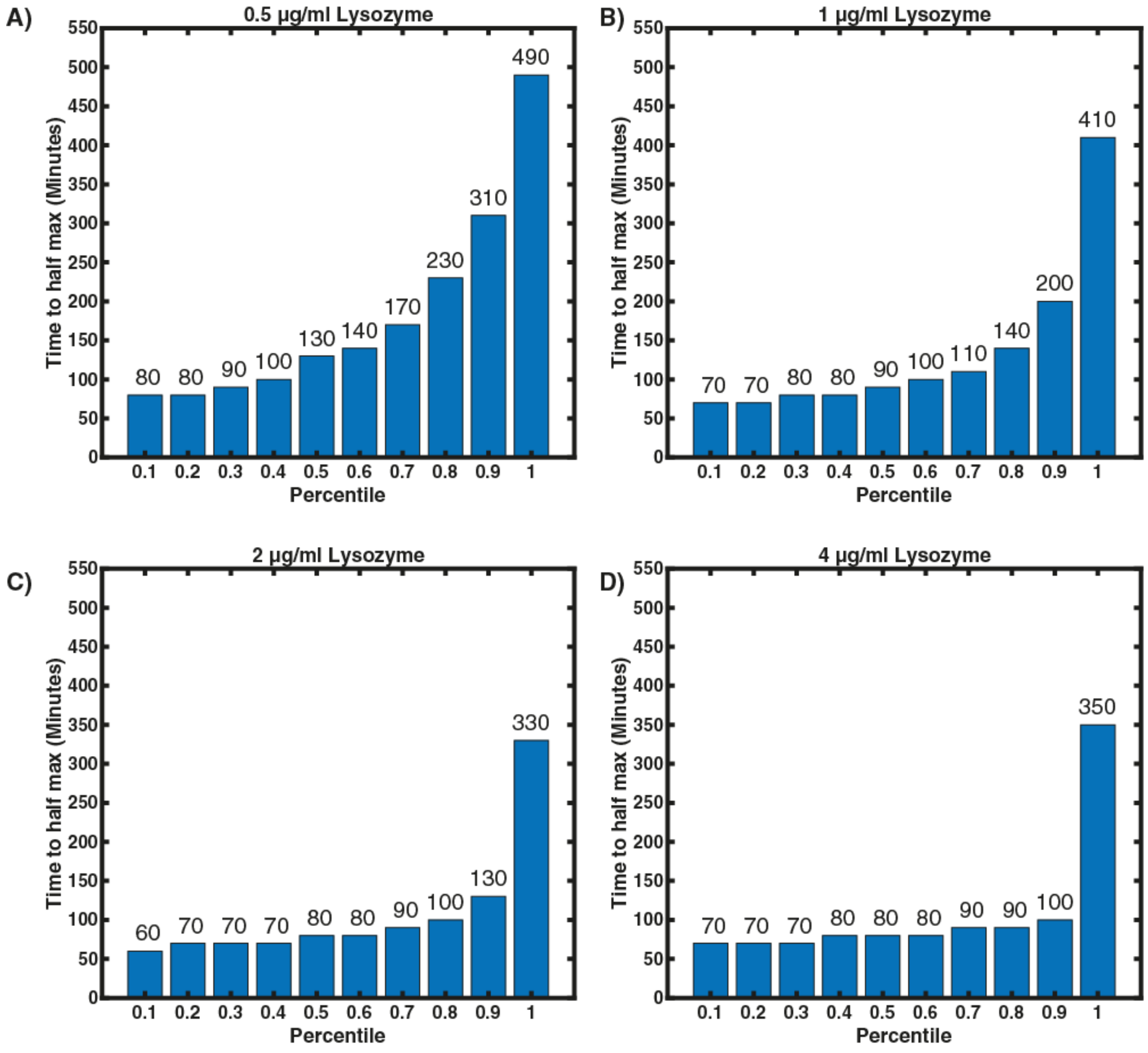

Appendix Figure S3. With increasing stress levels it takes less time for all cells to activate the σ^V pathway.

Each bar represents the maximum value of each 10th percentile of activation times. The activation time was calculated as the time between switching from SMM to lysozyme and the time point when then P$_{sigV}$-YFP passed its half maximum. The number above the bar indicates the maximum activation for the corresponding 10th percentile. The subfigures show the data for N number of cells from n biological repeats. (A) σ^V activation times in response to 0.5 µg/ml lysozyme (N=95, n=2). (B) σ^V activation times in response to1 µg/ml lysozyme

(N=109, n=2). (C) σ^V activation times in response to 2 µg/ml lysozyme (N=114, n=2). (D) σ^V activation times in response to 4 µg/ml lysozyme (N=100, n=2).

Data information: For more information on the number of repeats please see Appendix Table S5.

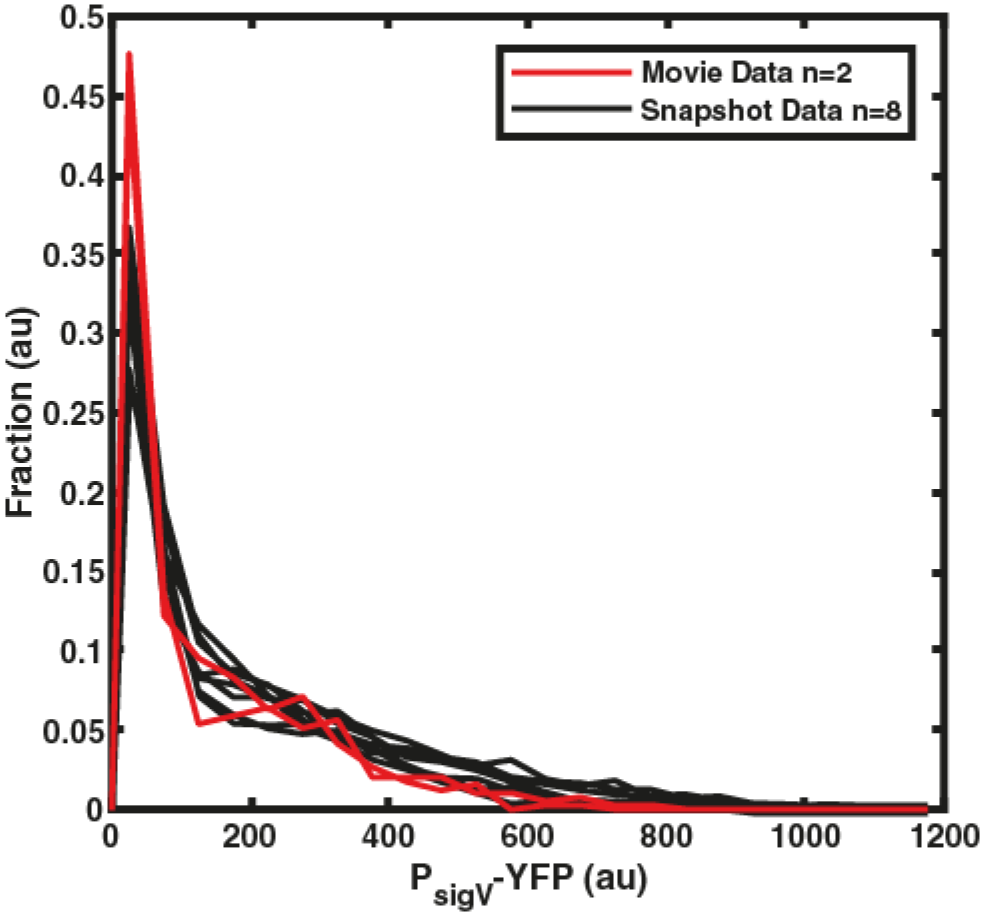

Appendix Figure S4. Cells grown in liquid culture and the mother machine both display long tailed σ^V activation distribution.

The black lines are the histograms of P_{sigV}-YFP fluorescence of cells grown in liquid culture (n=8 biological repeats with N>2600 cells each) and imaged with snapshots after being exposed to 1 µg/ml lysozyme for 30 min. The red lines are the histograms of P_{sigV}-YFP fluorescence of cells grown in the mother machine after being exposed to 1 µg/ml lysozyme for 30 min (n=2 with N>50 cells each).

Data information: For more information on the number of repeats please see Appendix Table S5.

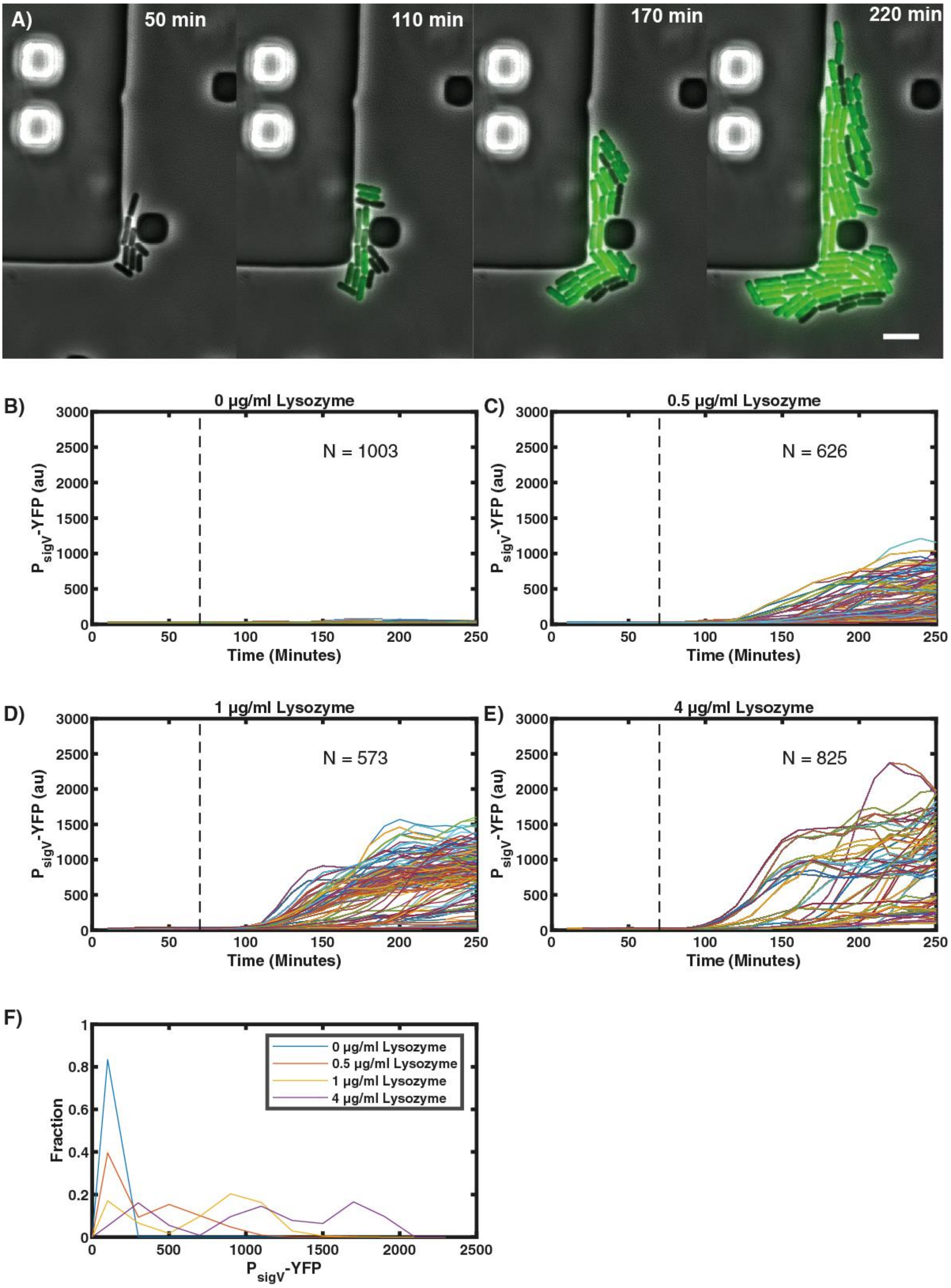

Appendix Figure S5. The observed heterogeneity in σ^V activation is not due to the geometry of the mother machine.

Cells grown in CellAsic bacteria chips also showed a heterogeneous activation of σ^V in response to lysozyme. In each subpanel stress was added after 60 min (dashed black line) and a line corresponds to a single-cell trace. Top panel (A) micrographs of cells grown in the CellAsic. Scale bar: 5 µm. (B) Single-cell traces in response to 0 µg/ml lysozyme. (C) Single-cell traces in response to 0.5 µg/ml lysozyme. (D) Single-cell traces in response to 1 µg/ml lysozyme. (E) Single-cell traces in response to 4 µg/ml lysozyme. (F) Histogram of single-cell P_{sigV}-YFP expression from the last frame of the movie. Only a fraction of cells respond to the lysozyme stress before the end of the movie and this fraction increases with increasing concentrations of lysozyme. The plotted data is from three biological repeats.

Data information: For more information on the number of repeats please see Appendix Table S5.

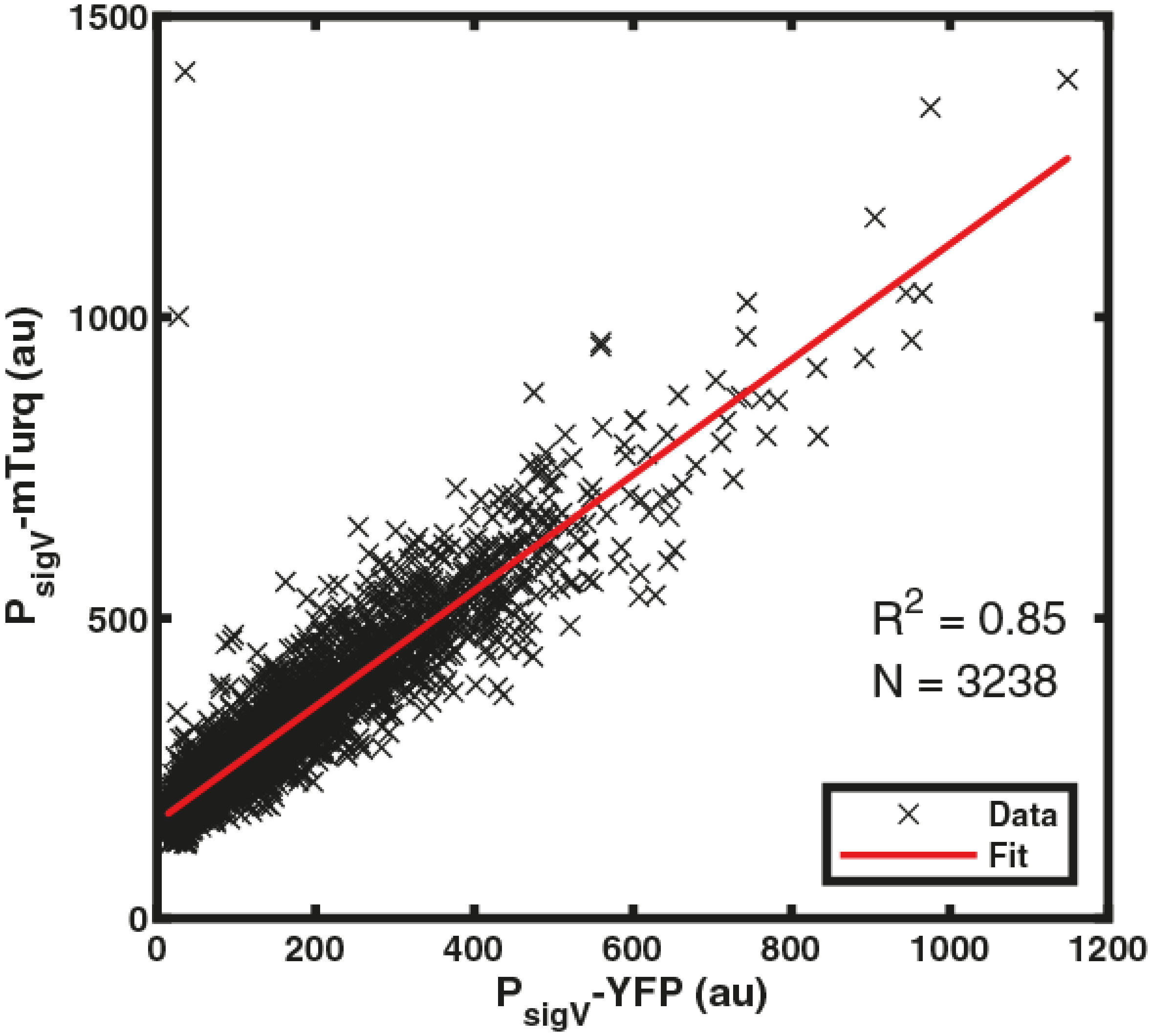

Appendix Figure S6. The observed heterogeneity in P_{sigV}-YFP activation in response to lysozyme reflects a heterogeneous σ^V activation and not intrinsic variability of the P_{sigV}-YFP promoter.

JLB213 (sacA::P_{sigV}-YFP Cm^R amyE::P_{sigV}-mTurq $Spec^R$) was exposed to 1 µg/ml lysozyme for 30 min and imaged with snapshots. Each data point corresponds to a single cell. Data from three biological repeats are plotted together (N=3238). The fit is a linear regression model.

Data information: For more information on the number of repeats please see Appendix Table S5.

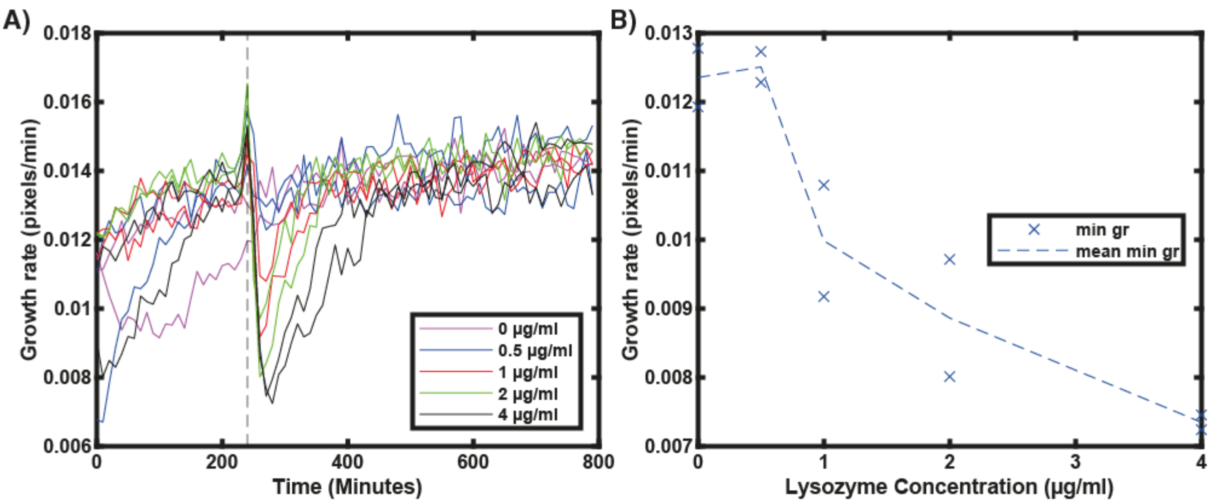

Appendix Figure S7. **Growth rate transiently decreases after the application of stress, with the minimal growth rate during the growth rate dip decreasing with increasing stress levels.**

(A) Single-cell growth rate traces for 0, 0.5, 1, 2 and 4 µg/ml lysozyme stress. The stress was added after 240 min at the black dashed line. After a spike in measured growth rate on application of stress (which could be due to changes in cell wall properties leading to an apparent cell size increase), growth rate transiently decreases. (B) Plot of minimal growth rate vs. lysozyme concentration.

Data information: For more information on the number of repeats please see Appendix Table S5.

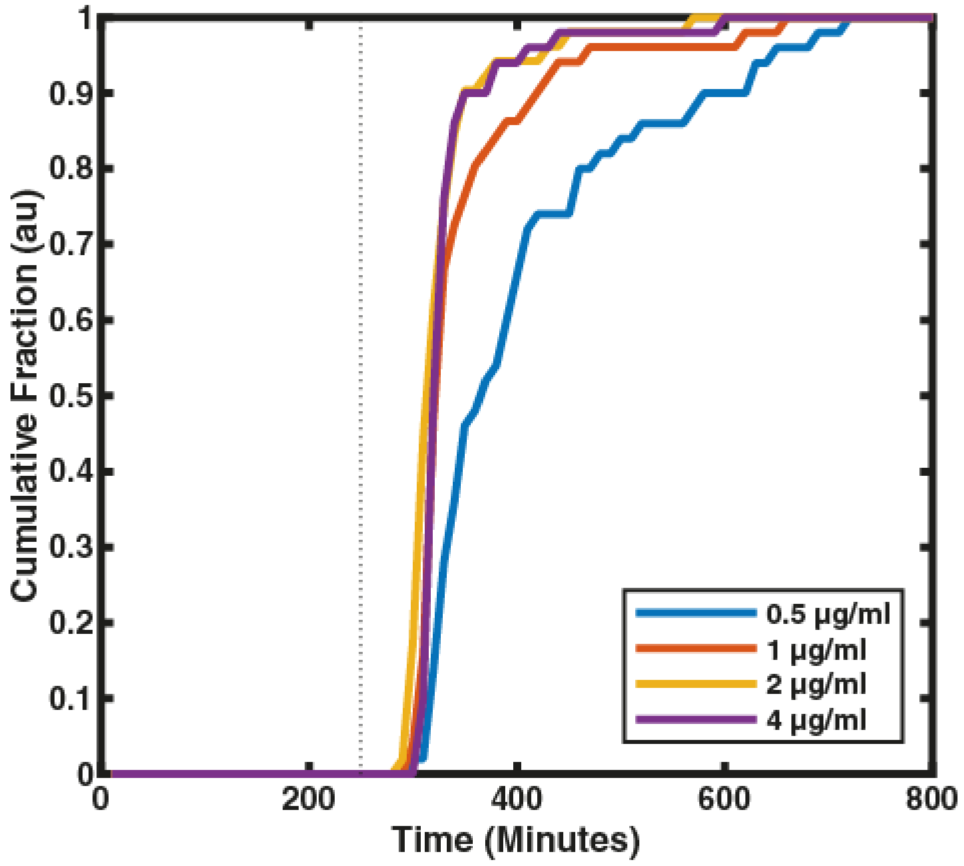

Appendix Figure S8. With increasing stress levels the heterogeneity in σ^V activation disappears.

The observed heterogeneity is reduced with increasing stress levels. Each line represents the time-dependent fraction of single cells to activate σ^V in response to lysozyme for N=~50 cells from one day. Data is a repeat experiment of Figure 1F.

Data information: For more information on the number of repeats please see Appendix Table S5.

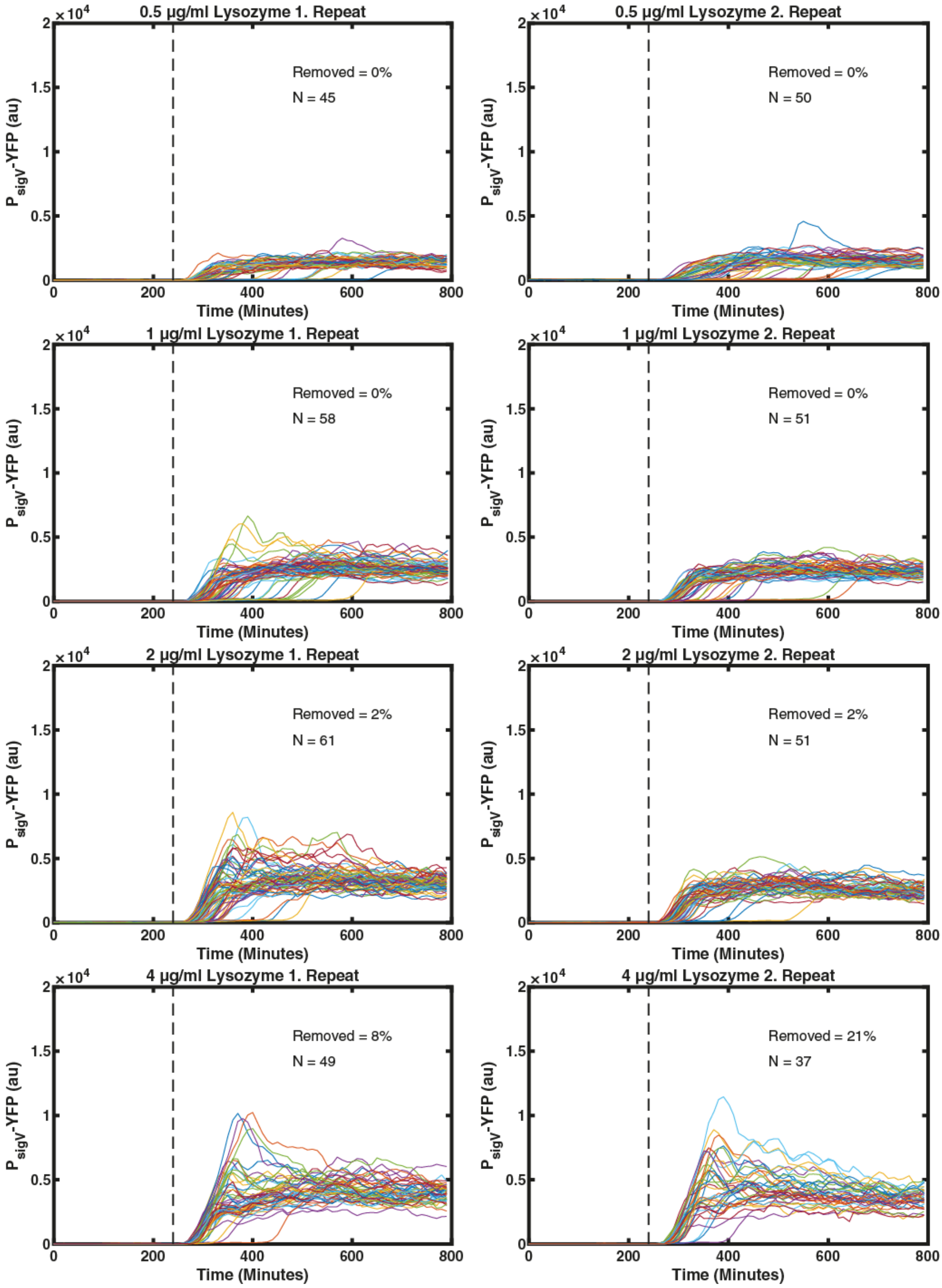

Appendix Figure S9. Removing all wide cells from analysis removes most cells which overshoot their σ^V activation.

The figure is the same as figure S1, but cells that are wider than unstressed cells are removed. In each subpanel a line corresponds to a single-cell trace of one mother cell in the mother machine. The stress was added after 240 min. (A&B) Single-cell traces in response to 0.5 µg/ml lysozyme. (C&D) Single-cell traces in response to 1 µg/ml lysozyme. (E&F) Single-cell traces in response to 2 µg/ml lysozyme. (G&H) Single-cell traces in response to 4 µg/ml lysozyme.

Data information: For more information on the number of repeats please see Appendix Table S5.

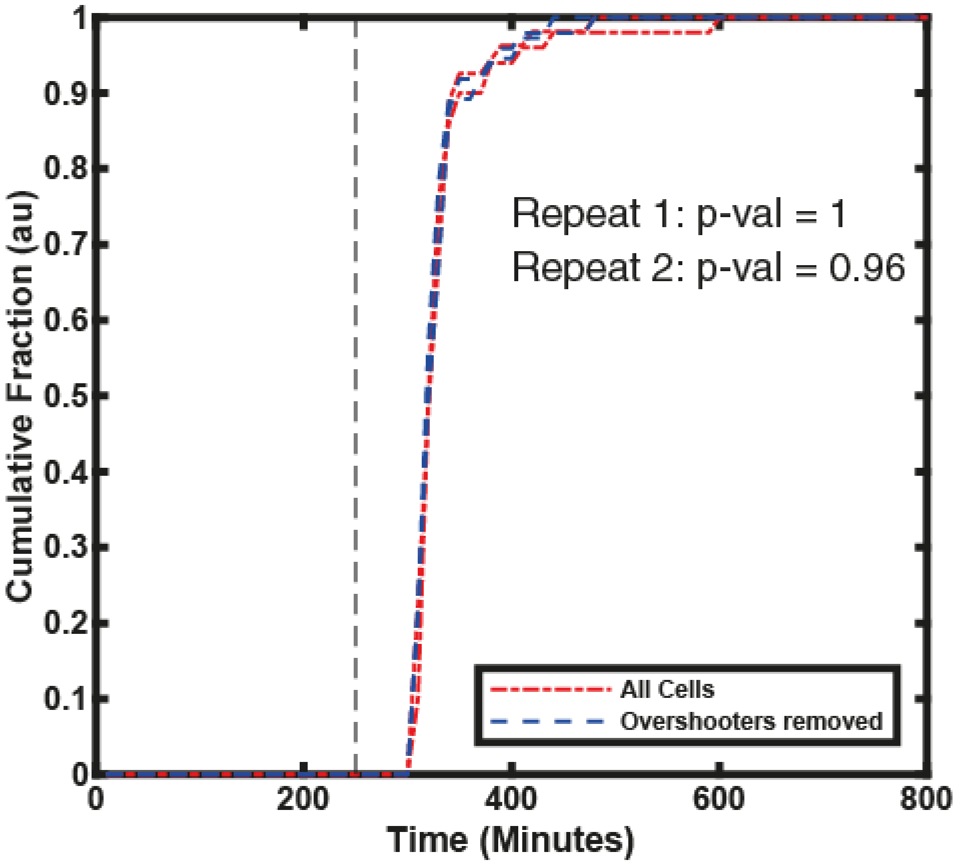

Figure S10. **Removing wide cells does not affect the observed heterogeneity.**

The cumulative fraction of cells with P_{sigV}-YFP values higher than the half maximum of their final values for 4 µg/ml lysozyme did not change when removing wide cells. The null hypothesis that the activation times of all cells and the activation times of cells without wide cells would have the same activation time distribution was not rejected using a Kolmogorov–Smirnov test. Only 4 µg/ml lysozyme (n=2) is shown as wide cells were mainly observed at this concentration.

Data information: For more information on the number of repeats please see Appendix Table S5.

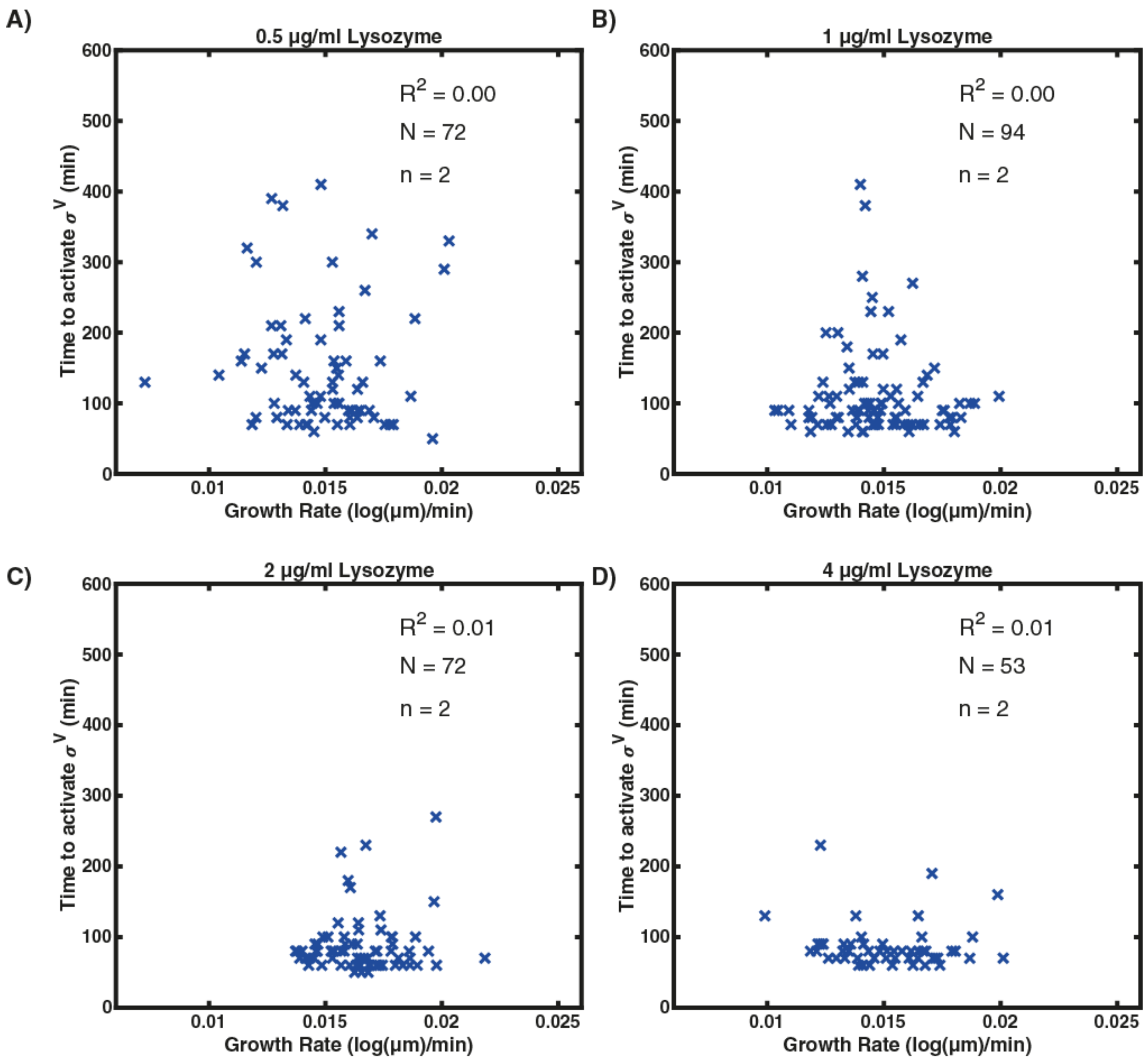

Figure S11. There is no correlation between growth rate and the activation time.

Figure plots the growth rate at the time of stress addition, against the time to P_{sigV}-YFP activation. Each cross corresponds to a single cell. (A) Growth rate vs. activation time for 0.5 µg/ml lysozyme (R^2=0.00). (B) Growth rate vs. activation time for 1 µg/ml lysozyme (R^2=0.00). (C) Growth rate vs. activation time for 2 µg/ml lysozyme (R^2=0.01). (D) Growth rate vs. activation time for 4 µg/ml lysozyme (R^2=0.01). Only growth rates during a cell cycle are plotted and not during a division. For more information see methods. Thus, N is smaller than in Appendix Figure S12.

Data information: For more information on the number of repeats please see Appendix Table S5.

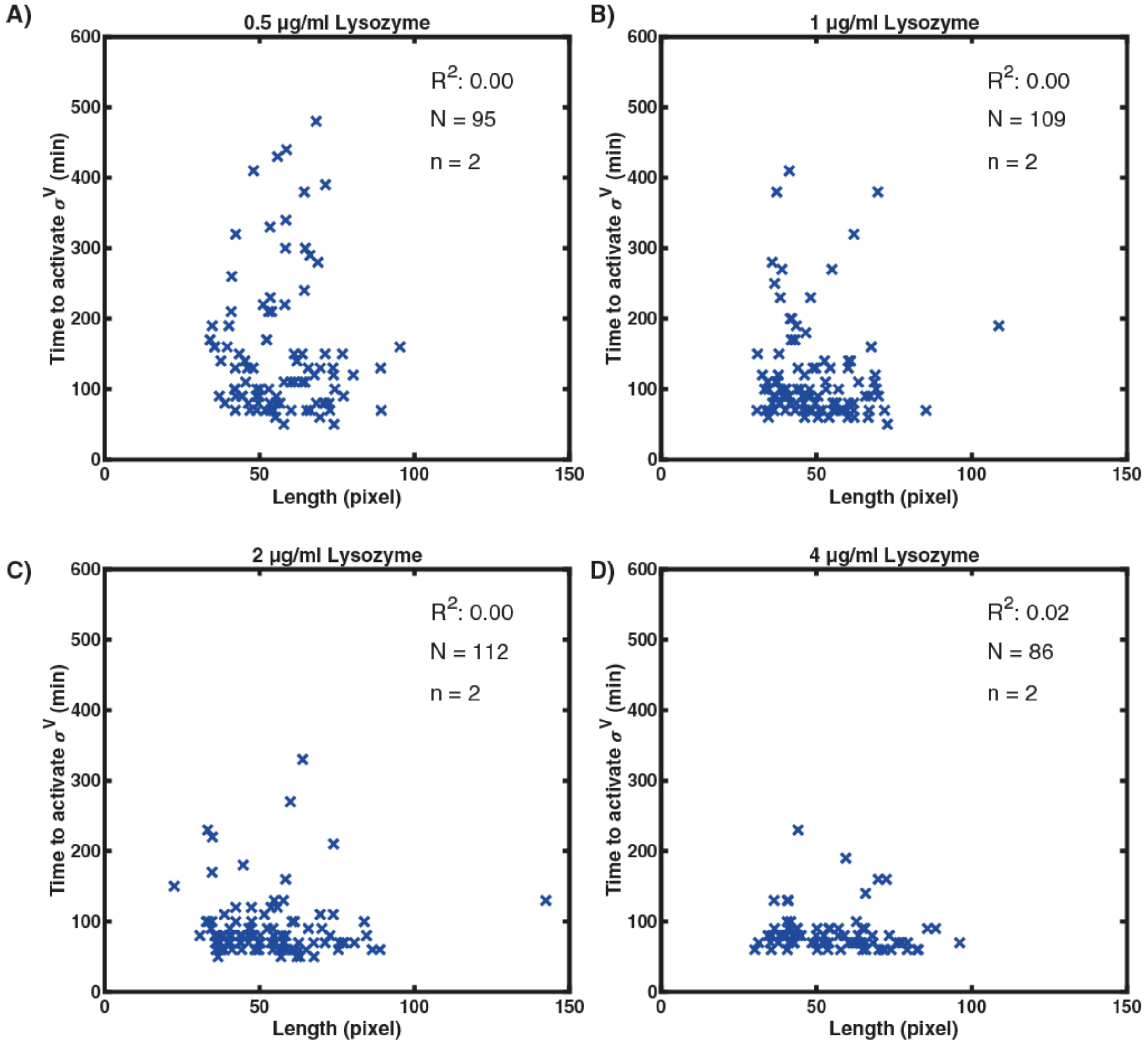

Appendix Figure S12. There is no correlation between cell length and the activation time.

Figure plots the cell length at the time of stress addition, against the time to P_{sigV}-YFP activation. Each cross corresponds to a single cell. (A) Cell length vs. activation time for 0.5 µg/ml lysozyme (R^2=0.00). (B) Cell length vs. activation time for 1 µg/ml lysozyme (R^2=0.00). (C) Cell length vs. activation time for 2 µg/ml lysozyme (R^2=0.00). (D) Cell length vs. activation time for 4 µg/ml lysozyme (R^2=0.01).

Data information: For more information on the number of repeats please see Appendix Table S5.

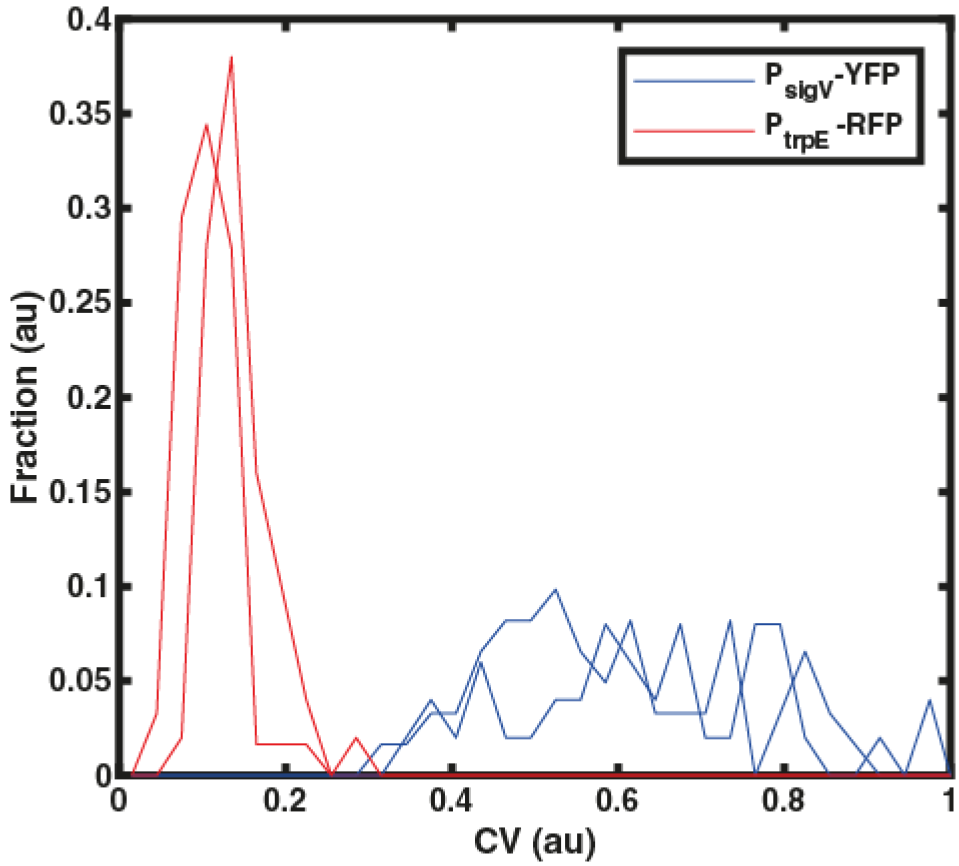

Appendix Figure S13. *sigV* expression displays variability even in absence of stress.

The CV for each mother cell across all time points (800 min) was calculated in the absence of lysozyme for P_{trpE}-RFP and P_{sigV}-YFP . The shown data is from two biological repeats with N ≥ 50 cells.

Data information: For more information on the number of repeats please see Appendix Table S5.

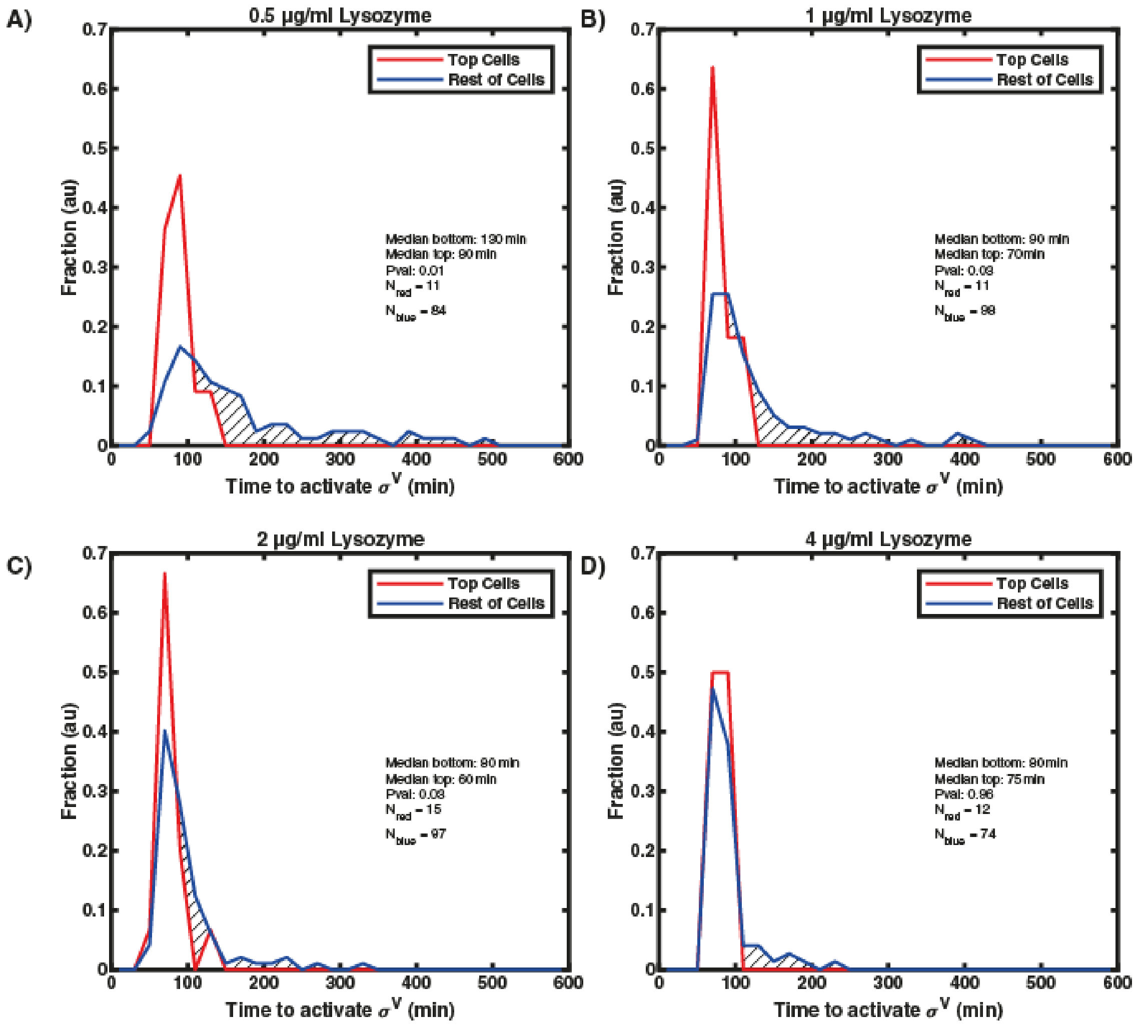

Appendix Figure S14. Cells with higher P_{sigV}-YFP levels before stress are more likely to activate σ^V instantaneously on lysozyme application.

Cells with a YFP fluorescence larger than the mean + SD are defined as Top Cells (red distribution). All other cells are captured in the blue distribution. The null hypothesis that cells with high P_{sigV}-YFP would have the same activation time distribution as cells with low P_{sigV}-YFP was rejected for 0.5 µg/ml, 1 µg/ml and 2 µg/ml lysozyme with a p-value of 0.05 using a Kolmogorov–Smirnov test. For more information see Methods. (A) Activation time distributions for 0.5 µg/ml lysozyme. (B) Activation time distributions for 1 µg/ml lysozyme. (C) Activation time distributions for 2 µg/ml lysozyme. (D) Activation time distributions for 4 µg/ml lysozyme. All shown data in the plots is from two biological repeats.

Data information: For more information on the number of repeats please see Appendix Table S5.

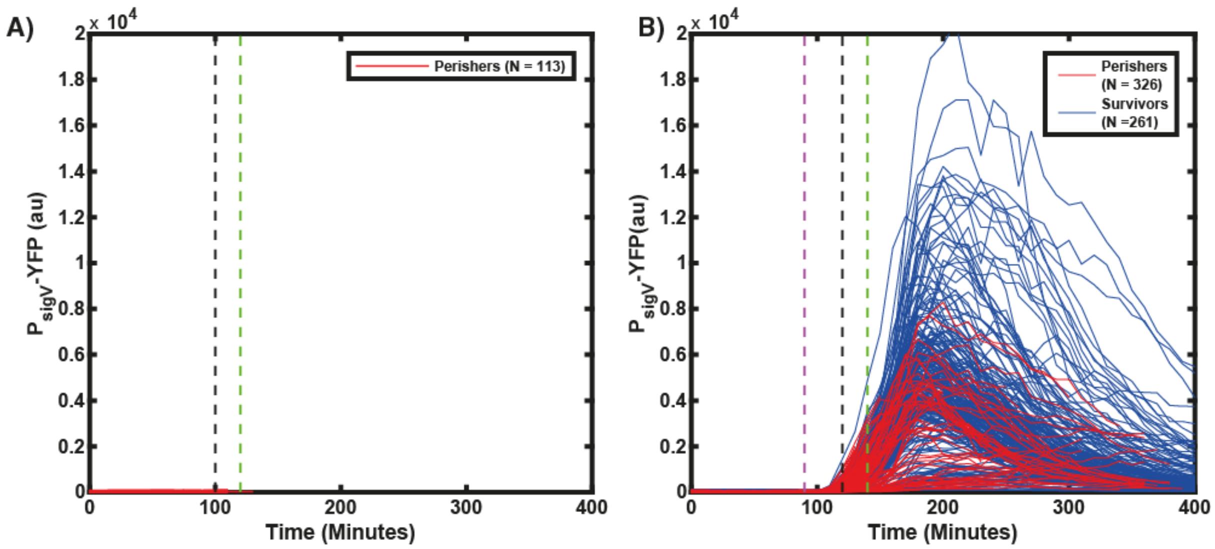

Appendix Figure S15. A sub-lethal stress improves survival to a subsequent lethal stress.

Cells were grown in the mother machine. Each line corresponds to the single-cell time trace of one mother cell. Perishers are defined as cells that die within 280 min (the end of the movie) after the addition of 20 µg/ml lysozyme while survivors continue to grow. (A) All cells die when exposed directly to high lysozyme concentrations. Cells were first grown in SMM and then at the black dashed line exposed to 20 µg/ml lysozyme. After 20 min the lysozyme stress was removed and the media was switched back to SMM (green dashed line). (B) Some cells survive the pulse of 20 µg/ml lysozyme having been exposed to 1 µg/ml lysozyme first. Cells were first grown in SMM before switching to 1 µg/ml lysozyme (magenta dashed line) for 30 min. This short exposure allows cells to activate their σ^V pathway and prepare the cell for the subsequent higher stresses. At the black dashed line the media was switched to 20 µg/ml lysozyme for 20 min before switching back to SMM (green dashed line).

Data information: For more information on the number of repeats please see Appendix Table S5.

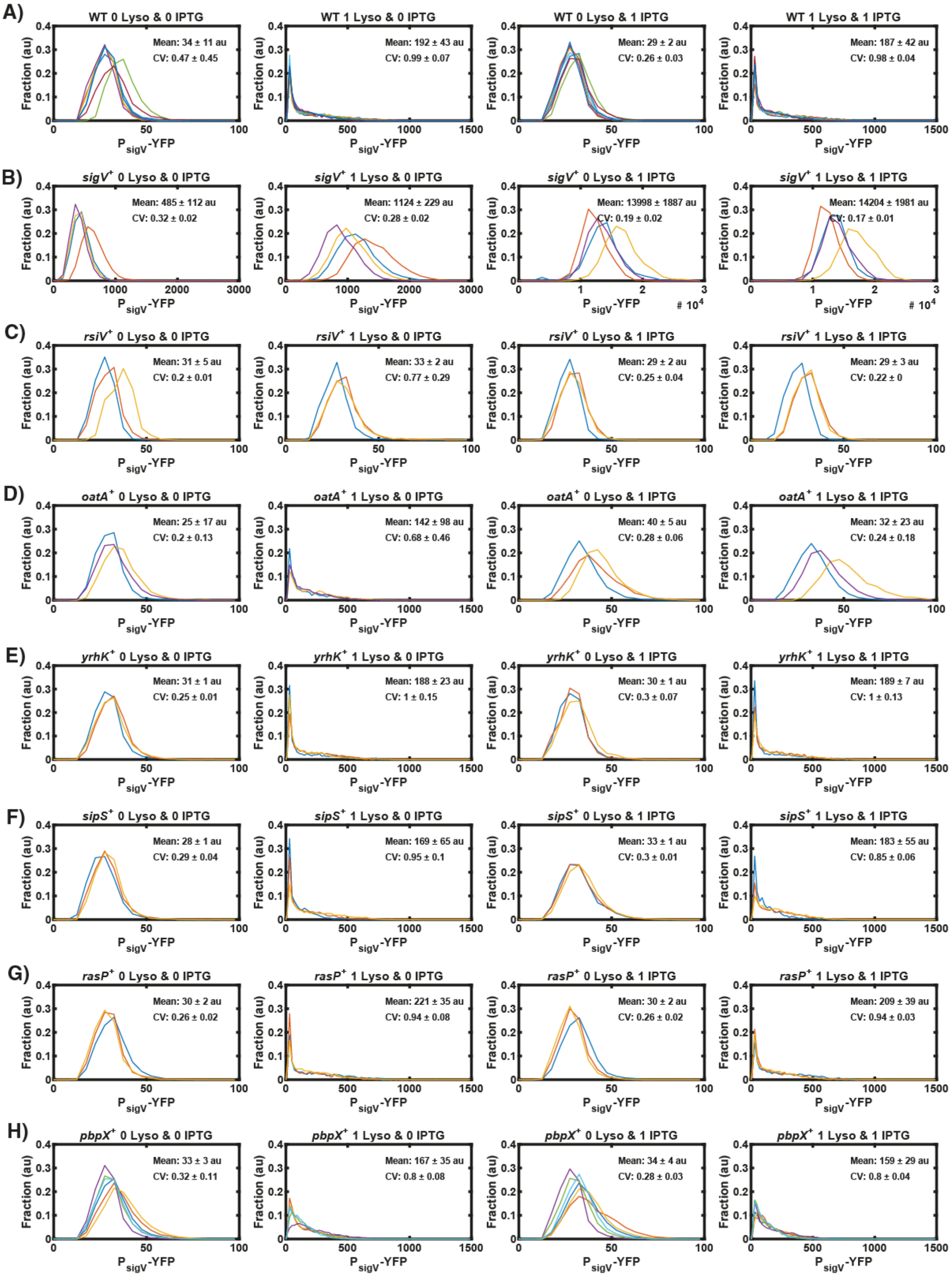

Appendix Figure S16. Overexpression of *yrhK, sipS, rasP* and *pbpX* have minor effect on the activation behaviour.

Each column corresponds to a different condition (0 mM IPTG and 0 µg/ml lysozyme, 0 mM IPTG and 1 µg/ml lysozyme, 1 mM IPTG and 0 µg/ml lysozyme, 1 mM IPTG and 1 µg/ml lysozyme). Each row has a different mutant: (A) WT (JLB130), (B) *sigV*$^+$ (JLB219), (C) *rsiV*$^+$ (JLB193), (D) *yrhK*$^+$ (JLB215), (E) *sipS*$^+$ (JLB216), (F) r*asP*$^+$ (JLB217), (G) *oatA*$^+$ (JLB218), (H) *pbpX*$^+$ (JLB191). Each coloured histogram corresponds to a different biological repeat (n ≥ 3) and each histogram consists of N >900 cells.

Data information: For more information on the number of repeats please see Appendix Table S5.

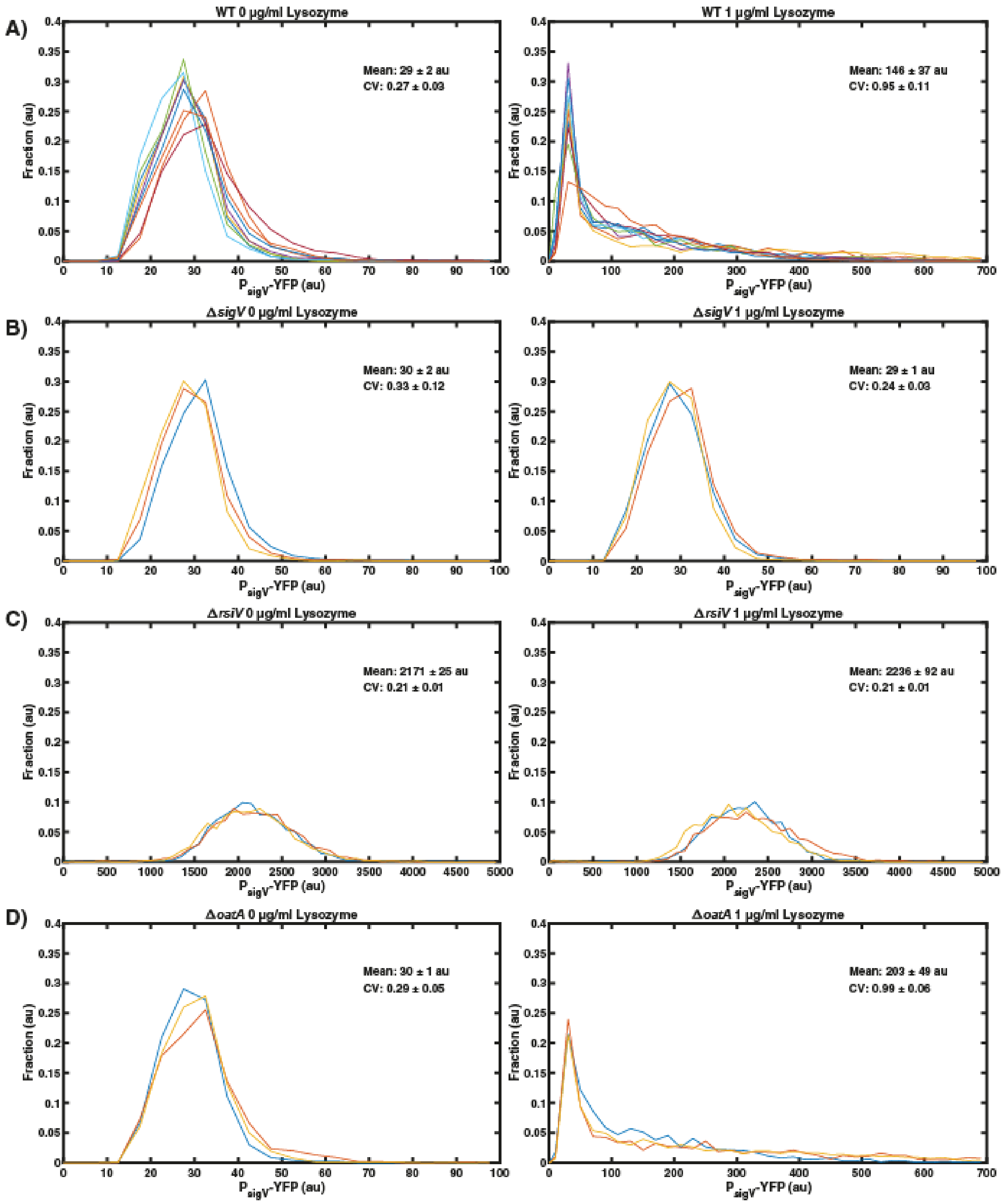

Appendix Figure S17. Deletion of *oatA* does not change the induction dynamics of P_{sigV}-YFP.

The first column corresponds to 0 µg/ml lysozyme and the second column corresponds to 1 µg/ml lysozyme. Each row is a different deletion mutant: (A) WT (JLB130), (B) Δ*sigV*

(JLB154), (C) Δ*rsiV* (JLB208), (D) Δ*oatA* (JLB156). Each coloured histogram corresponds to a different biological repeat.

Data information: For more information on the number of repeats please see Appendix Table S5.

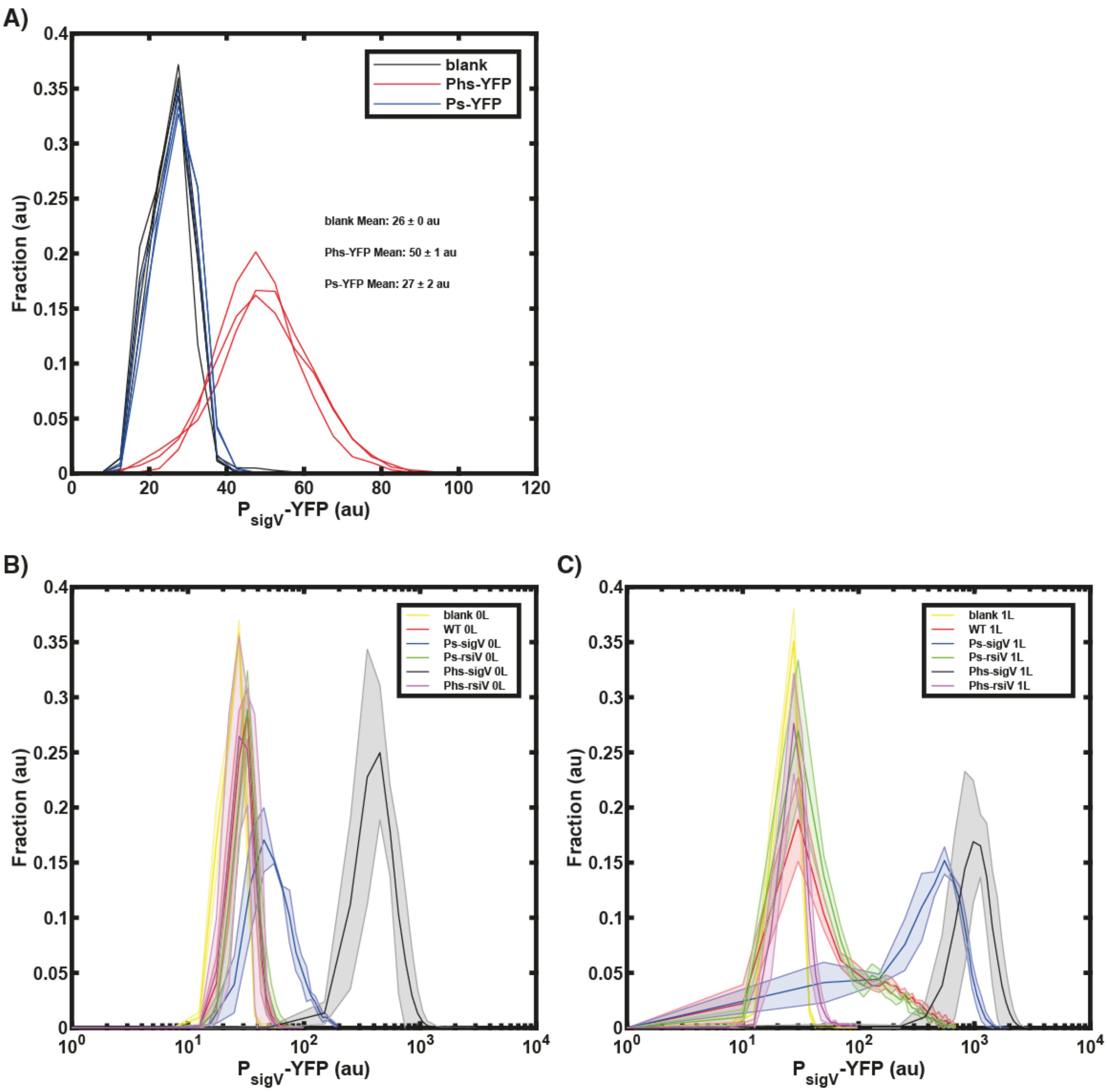

Appendix Figure S18. The *sigV* activation dynamics are sensitive to *sigV* and *rsiV* baseline levels.

A) The baseline expression of P_{spank}-YFP is almost the same as a strain without a YFP reporter. Baseline expression of blank (JLB54), $P_{hyperspank}$-YFP (JLB244) and P_{spank} -YFP (JLB242) with 0 uM IPTG are shown. Each line is a biological repeat. B&C) The *sigV* activation dynamics are sensitive to σ^V and RsiV baseline levels. The histograms for blank (JLB54), WT (JLB130), P_{spank}-*sigV* (JLB209), P_{spank}-*rsiV* (JLB196), $P_{hyperspank}$-*sigV* (JLB219) and $P_{hyperspank}$-*rsiV* (JLB193) are shown for 0 µg/ml lysozyme (B) and 1 µg/ml Lysozyme (C).

In B & C) the solid lines are the mean histograms and the shaded areas are the standard deviation of the mean.

Data information: For more information on the number of repeats please see Appendix Table S5.

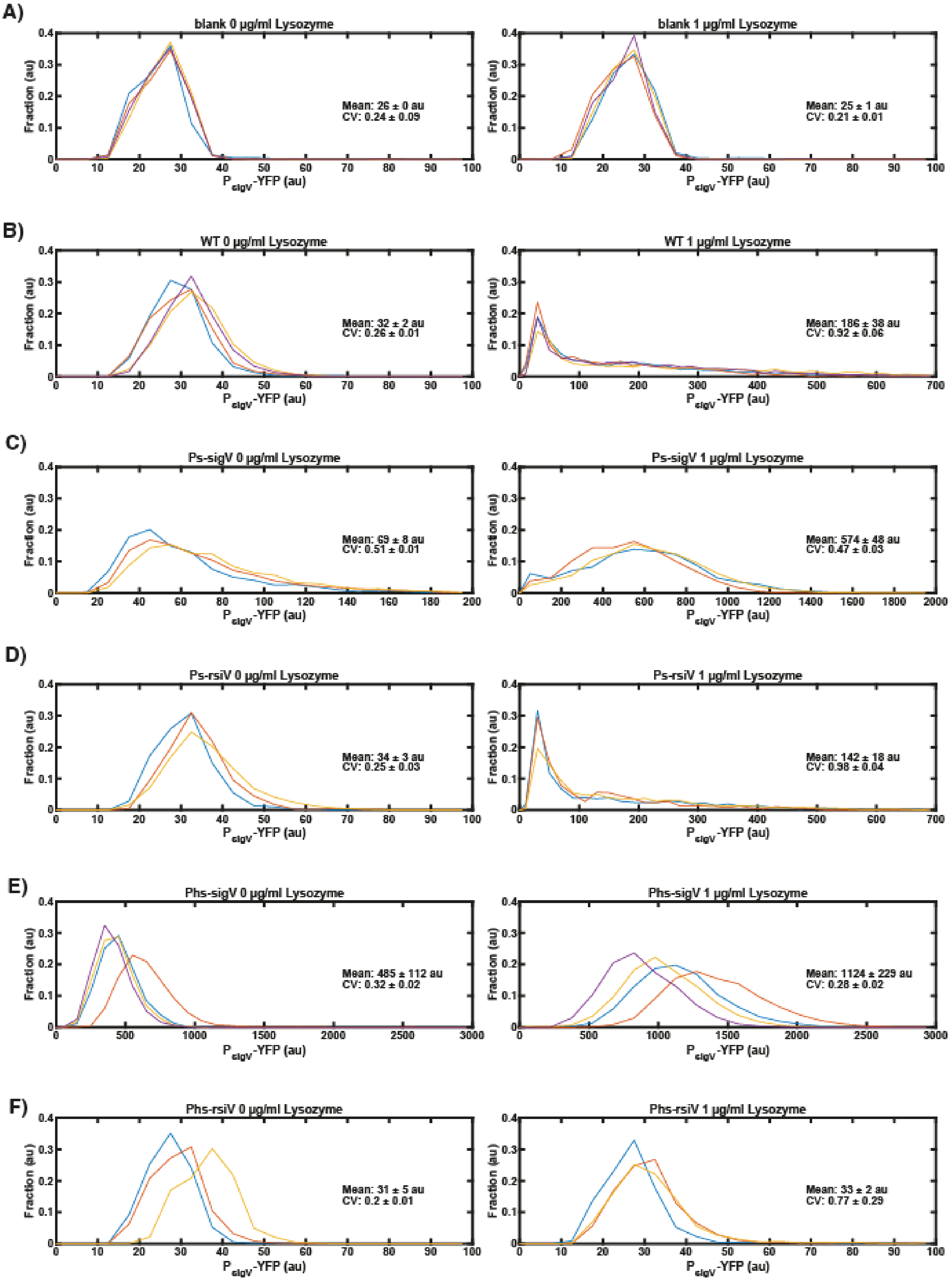

Appendix Figure S19. The *sigV* activation dynamics are sensitive to σ^V and RsiV baseline levels.

The first column corresponds to 0 µg/ml lysozyme and the second column corresponds to 1 µg/ml lysozyme. Each row is a different mutant: (A) blank (JLB54), (B) WT (JLB130), (C) P_{spank}-*sigV* (JLB209), (D) P_{spank}-*rsiV* (JLB196), (E) $P_{hyperspank}$-*sigV* (JLB219), (F) $P_{hyperspank}$-*rsiV* (JLB193). Each coloured histogram corresponds to a different biological repeat.

Data information: For more information on the number of repeats please see Appendix Table S5.

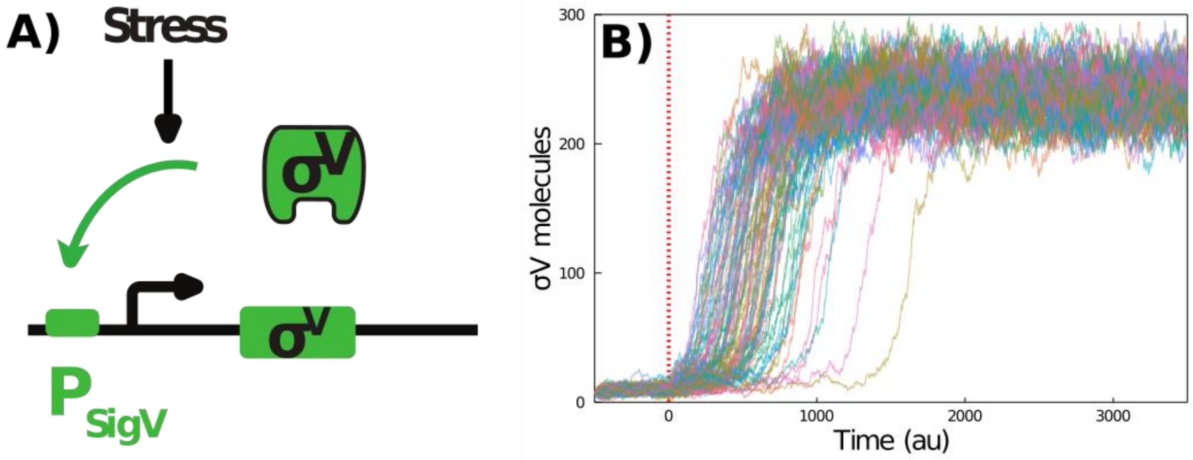

Appendix Figure S20. A minimal model of only σ^V self-activation can explain the heterogeneous activation.

(A) In addition to modelling the σ^V network with both σ^V and RsiV, we modelled it as a minimal self-activation loop with only σ^V activating its own production. (B) Using only this model we were able to recreate the heterogeneous activation of the system. However, this model could not be compared to the RsiV mutants (as in Figure 5), hence we decided to continue with only the full σ^V /RsiV model. The minimal model consists of the single differential equation $d\sigma/dt = v_0 + L{*}v{*}(\sigma^n)/(\sigma^n + K^n) - d{*}\sigma$, where L is the stress input (in (B), at time 500, changed from 0 to 1). Parameter values are (v_0,v,K,n,d) = (0.1 Molecules·min^{-1}, 2.5 Molecules·min^{-1}, 75.0 Molecules, 2, 0.01 min^{-1}).

Data information: For more information on the number of repeats please see Appendix Table S5.

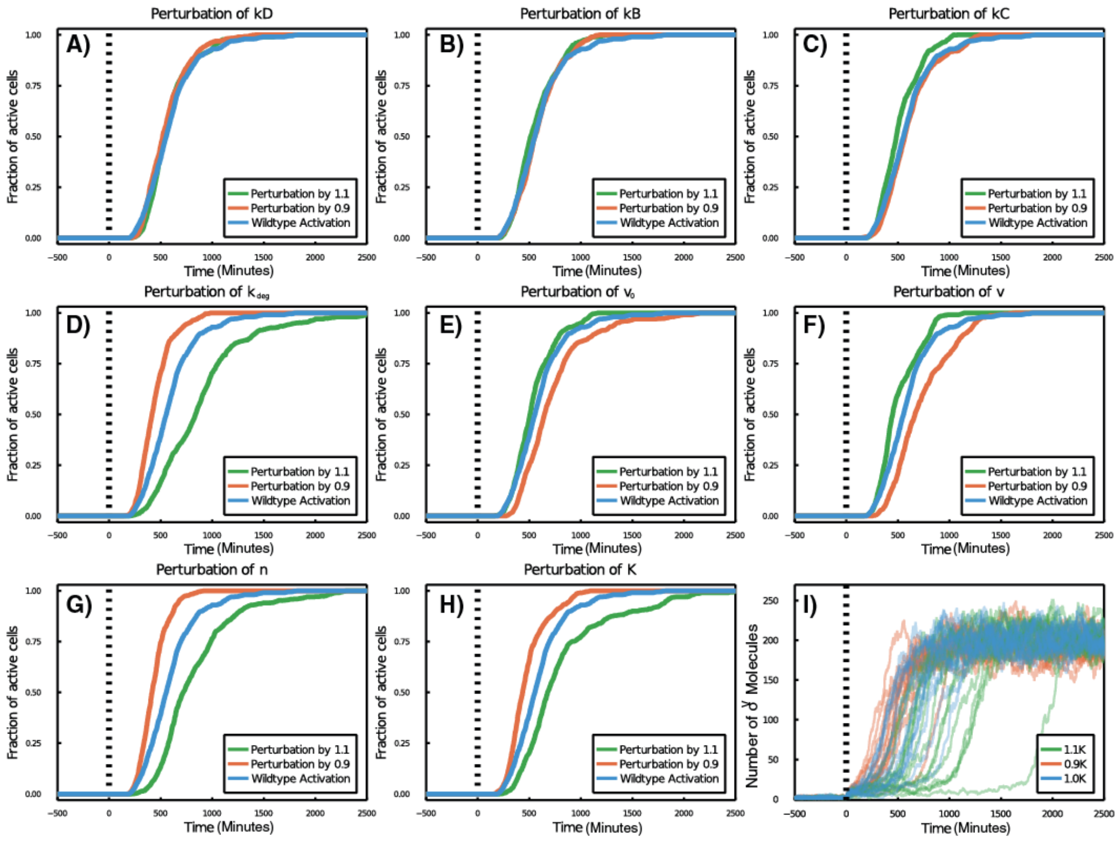

Appendix Figure S21. The model behaviour is preserved under parameter perturbations.

(A-H) Each parameter of the system is perturbed by 10%, and the effect on the cumulative activation diagram is shown (the parameter *L* is not perturbed, since this is equivalent to perturbing *kC*). Perturbing *K* has a relatively large effect on the system, however, the normal activation behaviour is still exhibited, as seen in (I), which contains 15 trajectories for the activation of a wild type system (blue), a system where *K* has been reduced by 10% (red) and increased by 10% (green). The heterogeneous activation behaviour is preserved, and the parameter perturbation simply tunes the activation.

Data information: For more information on the number of repeats please see Appendix Table S5.

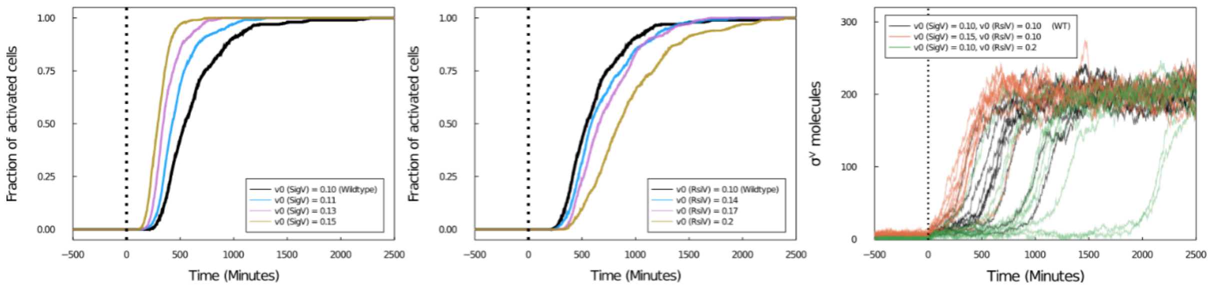

Appendix Figure S22. Increased baseline activity of either system component modulates activation heterogeneity.

(A) For a modified model, where the baseline expression of production of σ^V is increased, the heterogeneity of the activation is reduced (reduction in heterogeneity increases with increased baseline expression) (N=100 for each baseline expression level). (B) If instead the baseline production of RsiV is increased, the activation heterogeneity also increases (N=100 for each baseline expression level). (C) Example trajectories for the wild type system, as well as for two systems, with increased baseline expression rate of σ^V or RsiV, respectively (N=10 for each of the three cases).

Data information: For more information on the number of repeats please see Appendix Table S5.

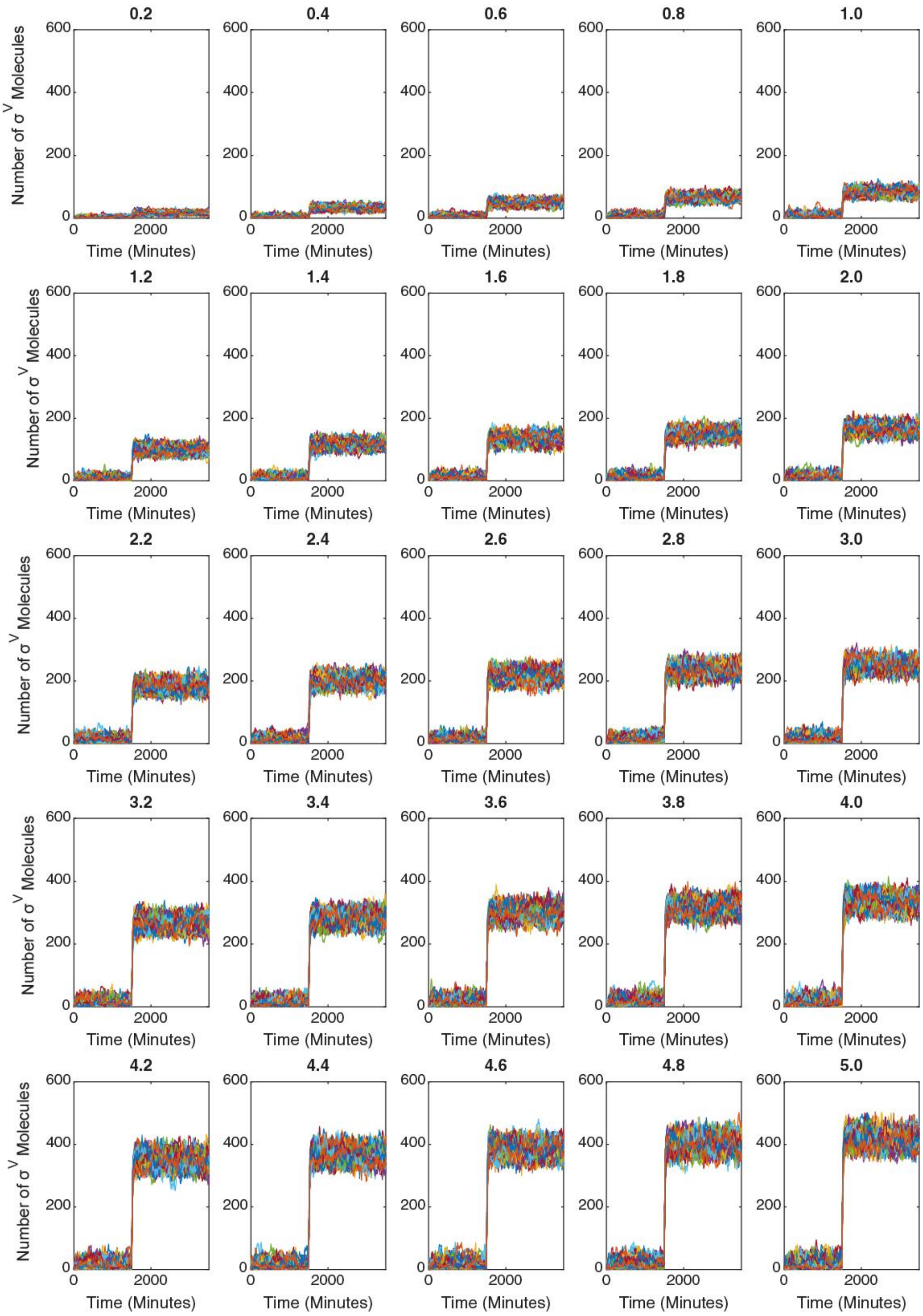

Appendix Figure S23. The *sigV* feedback loop increases the dynamic range of the circuit in the model.

The shown simulations were used to calculate the fold change in Figure EV5A. Title above each plot indicates the specific induction strength. Induction occurs at time point 1500.

Data information: For more information on the number of repeats please see Appendix Table S5.

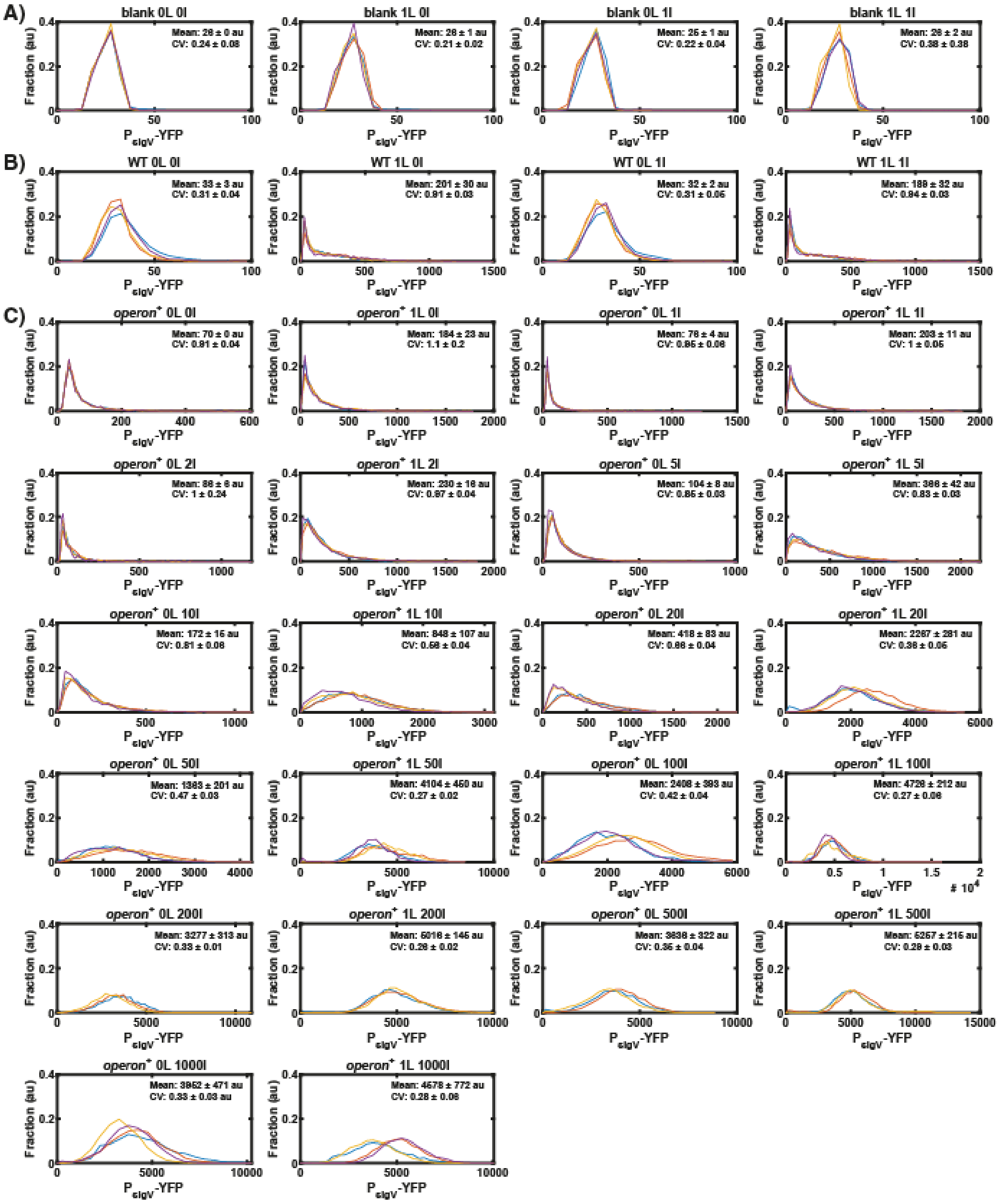

Appendix Figure S24. **The *sigV* feedback loops increase the dynamic range of the circuit.**

(A & B) Histograms of no *sigV* reporter (blank) and WT for 0 mM IPTG and 0 µg/ml lysozyme (0L & 0I), 0 mM IPTG and 1 µg/ml lysozyme (1L & I), 1 mM IPTG and 0 µg/ml lysozyme (0L & 1I), 1 mM IPTG and 1 µg/ml lysozyme (0L & 1I). (C) Histograms of the feedback-broken strain, Operon^{+} (Δ*sigVrsiVoatAyrhK* P_{spank}-*sigVrsiVoatAyrhK*), are shown for a range of

concentration of IPTG ranging from 0 uM (0I) to 1000uM (1000I) with (1L) and without lysozyme stress (0L). Each coloured histogram corresponds to a different biological repeat.

Data information: For more information on the number of repeats please see Appendix Table S5.

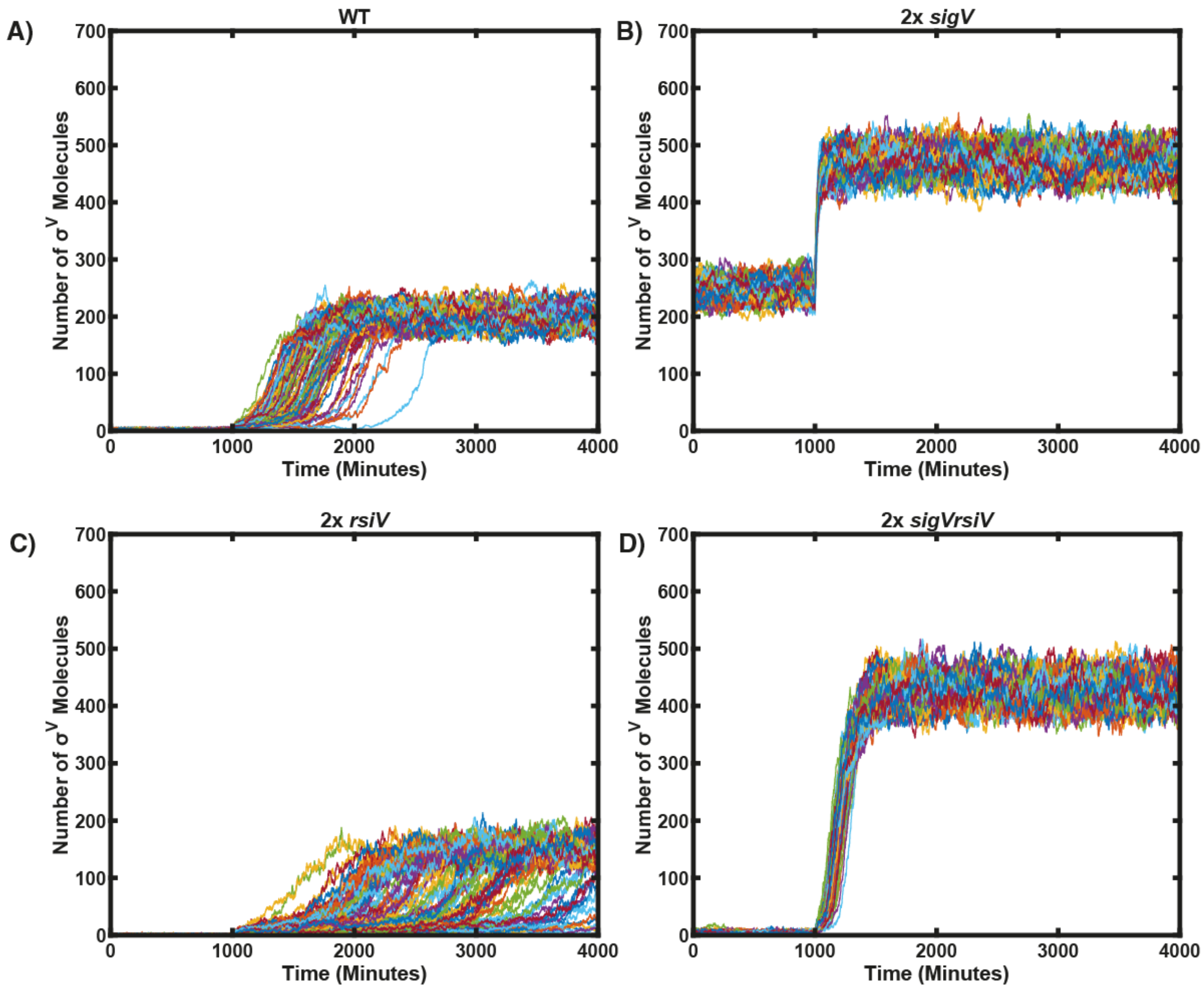

Appendix Figure S25. The model predicts a genetic tunability of the heterogeneity in σ^V response to lysozyme.

In these model simulations a second copy of either *sigv*, *rsiV* or both *sigVrsiV* was introduced (N=100). Stress is added at time 1000. (A) WT, (B) 2x *sigV,* (C) 2x *rsiV,* (D) 2x *sigVrsiV.*

Data information: For more information on the number of repeats please see Appendix Table S5.

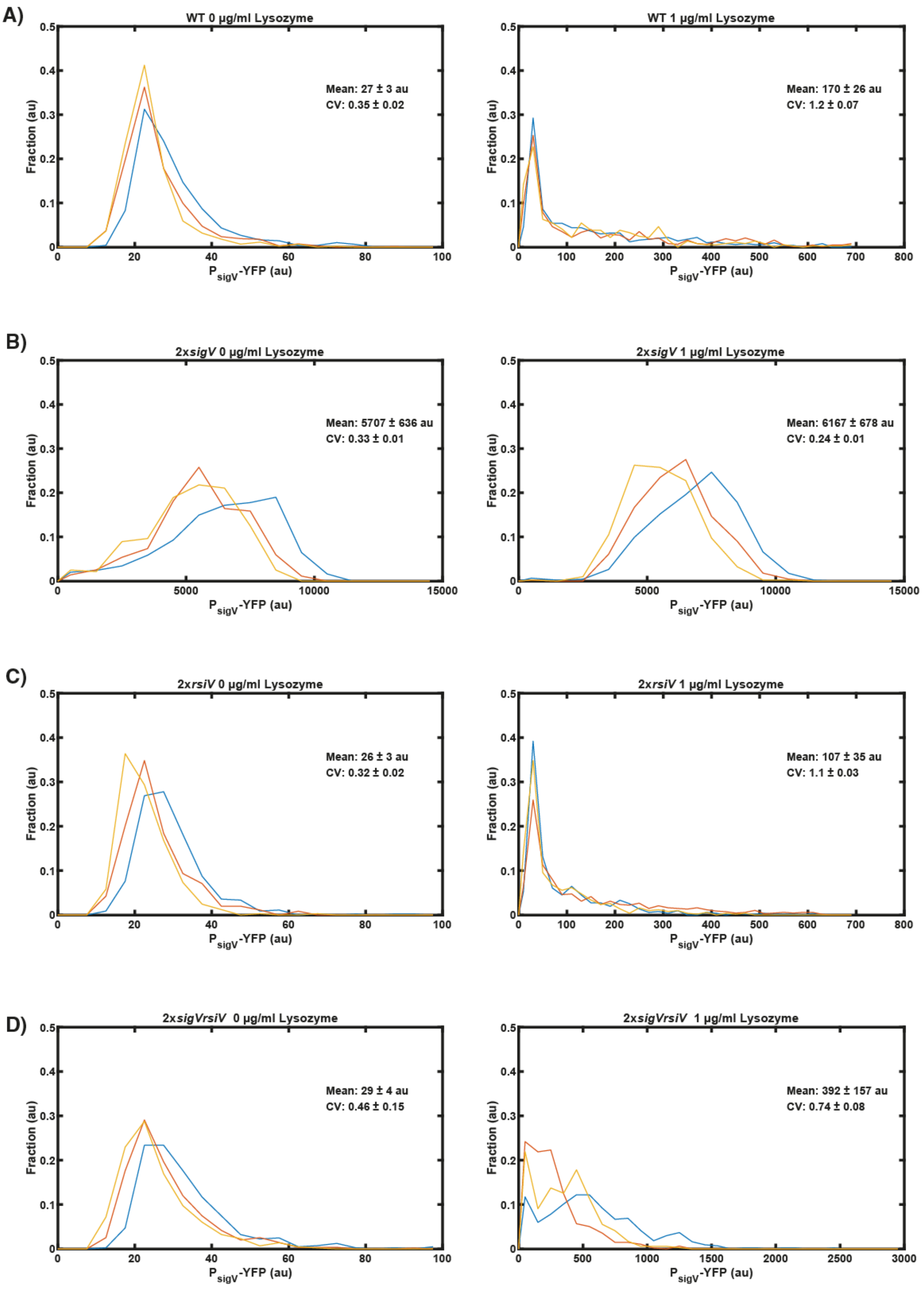

Appendix Figure S26. Snapshots confirm that the σ^V response to lysozyme is genetically tunable.

The first column corresponds to 0 μg/ml lysozyme and the second column corresponds to 1 μg/ml lysozyme. Each row is a different mutant: (A) WT (JLB130), (B) 2x *sigV* (JLB210), (C) 2x *rsiV* (JLB212), (D) 2x *sigVrsiV* (JLB211). Each coloured histogram corresponds to a different biological repeat.

Data information: For more information on the number of repeats please see Appendix Table S5.

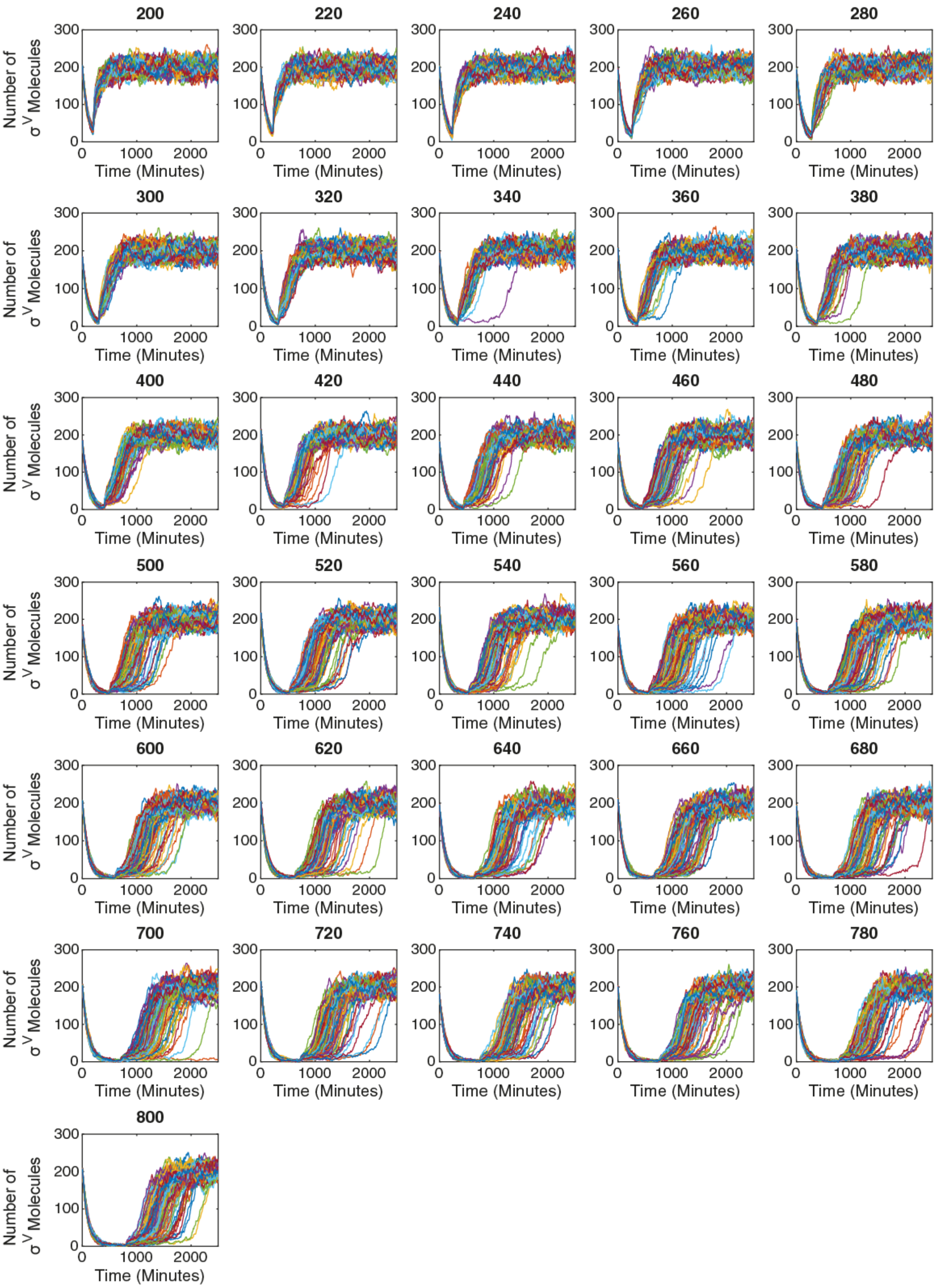

Appendix Figure S27. The memory effect decreases with increasing delay after stress removal in the model.

Each line corresponds to one simulated cell. At time 0 the stress was removed and reapplied at time points 200 - 800 (N=100 for each panel).

Data information: For more information on the number of repeats please see Appendix Table S5.

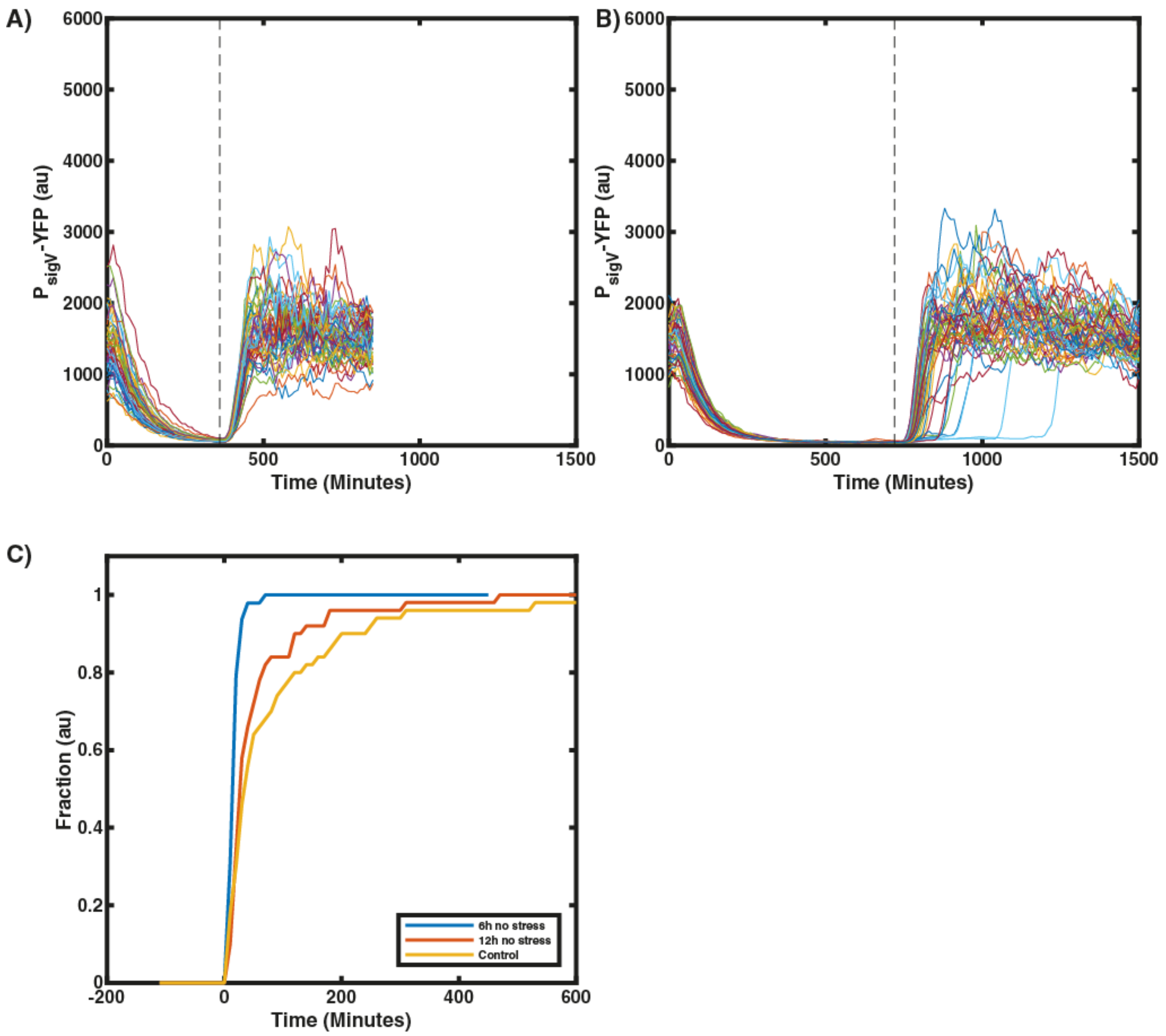

Appendix Figure S28. Repeat experiment also shows a memory in σ^V after a stress break.

The stress was removed at time 0 min and reapplied after 6h or 12h. Each line corresponds to one mother cell. (A) 6h stress break. (B) 12h stress break. (C) This figure shows the fraction of cells with P_{sigV}-YFP larger than their half maximum.

Data information: For more information on the number of repeats please see Appendix Table S5.

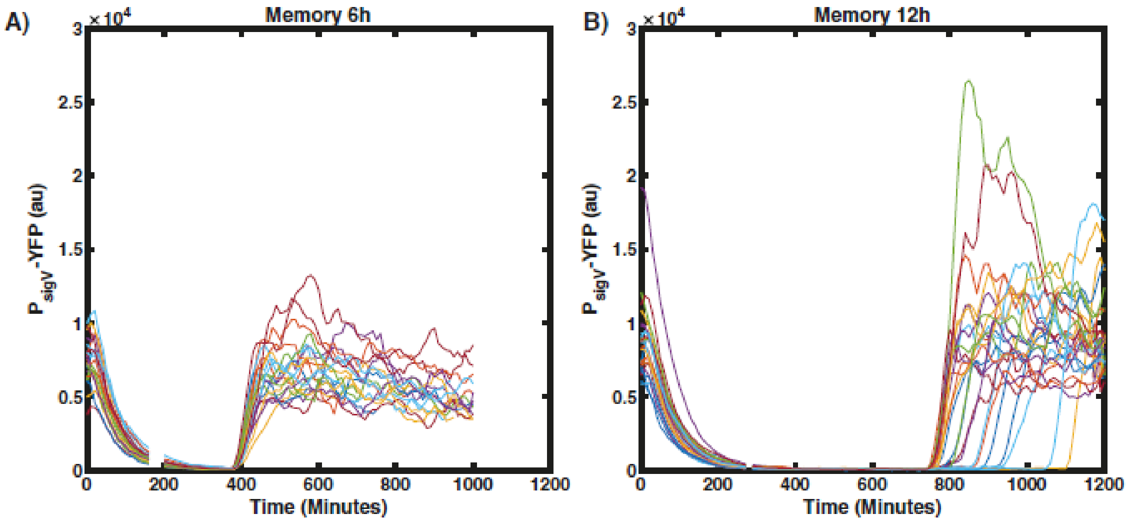

Appendix Figure S29. The memory dynamics are independent of the design of the mother machine microfluidic device.

JLB130 was grown in the mother machine microfluidic device with a design from the Paulsson lab (Norman et al. 2013) rather than our standard mother machine design based on the chip from the Jun lab (Wang et al. 2010). (A) After a 6h long break in stress all cells responded homogeneously to the reapplication of 1 µg/ml lysozyme (N=20). (B) Cells grown in the mother machine were exposed to a 12h long break in stress. After the break in stress the heterogeneity in σ^V activation reappeared (N=26). The gaps in the traces (before 200 min) correspond to frames where the cells were out of focus. (n=1).

Data information: For more information on the number of repeats please see Appendix Table S5.

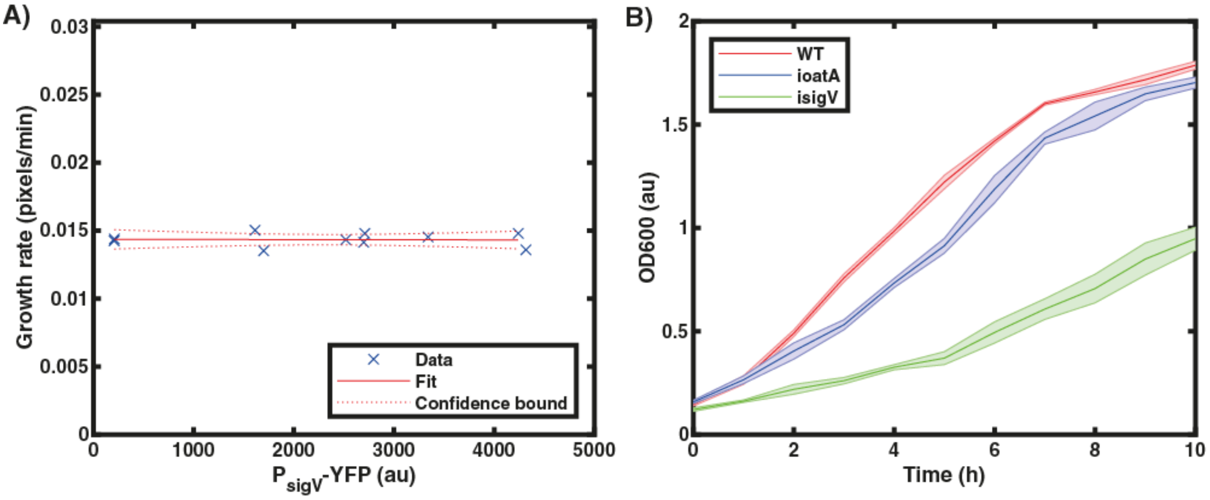

Appendix Figure S30. Constitutive expression of *sigV* reduces growth.

A) In the mother machine movies high levels of *sigV* expression do not seem to affect the growth rate. The data was fitted to a linear regression model. The confidence bound has a 95% confidence level. B) The growth curves in liquid culture when overexpressing *sigV* or *oatA* show a reduced growth rate compared to the WT. 1mM of IPTG was added to the cultures 1 h before the start of the experiment (for more details see methods). The solid lines are the mean growth curves and the shaded areas are the standard deviation of the mean.

Data information: For more information on the number of repeats please see Appendix Table S5.

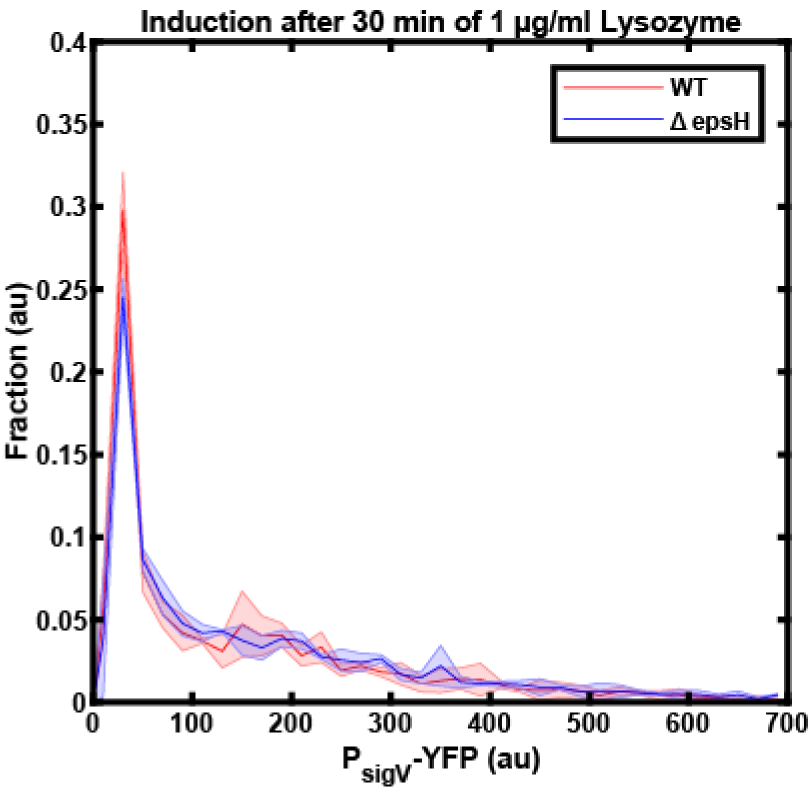

Appendix Figure S31. WT and ΔepsH have the same P*sigV*-*YFP* activation in snapshots.

WT (JLB130) and ΔepsH (JLB221) were grown in SMM with 1 µg/ml lysozyme for 30 min and then investigated with snapshots (n=3). The solid lines are the mean histograms and the shaded areas are the standard deviation of the mean.

Data information: For more information on the number of repeats please see Appendix Table S5.

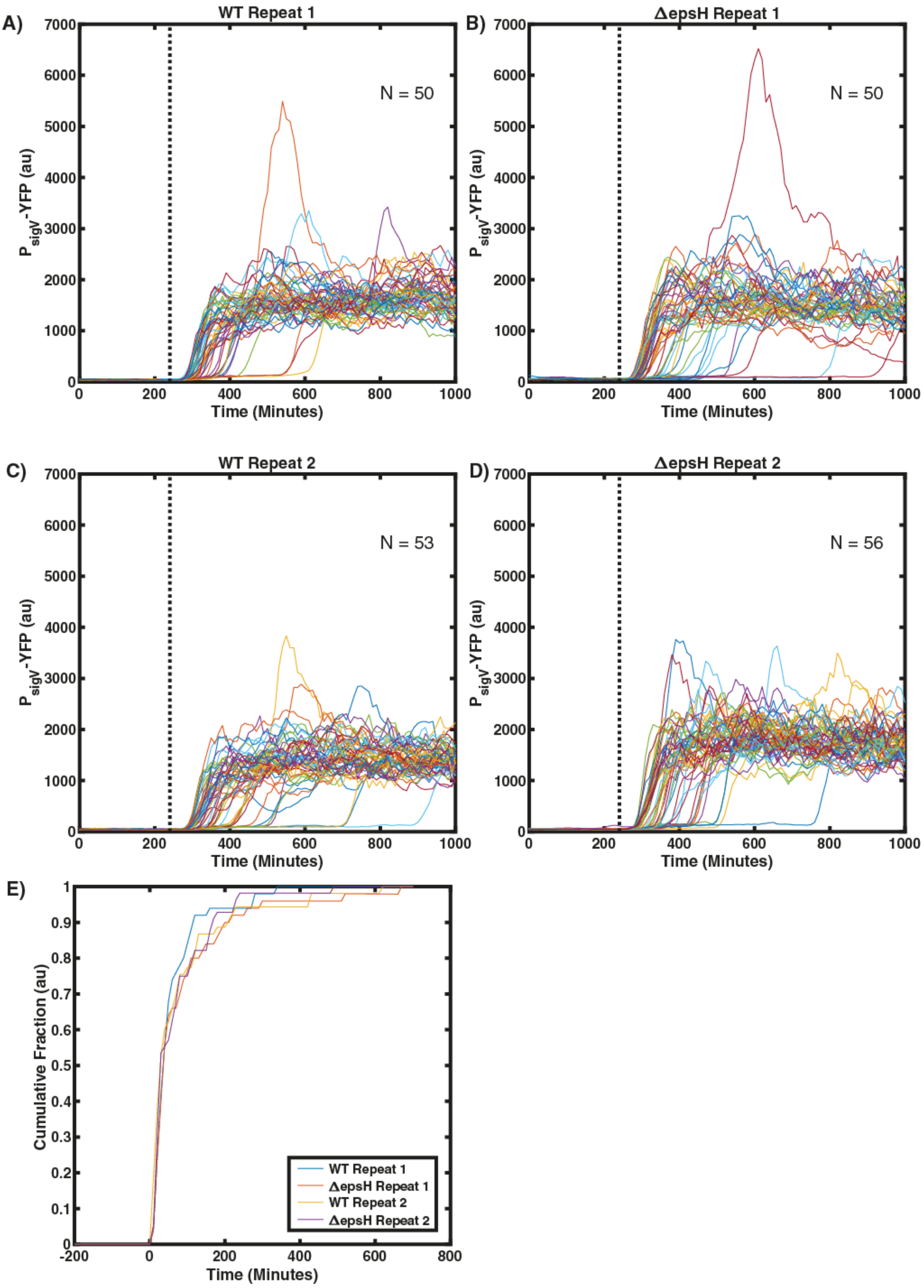

Appendix Figure S32. WT and ΔepsH have similar P_{sigV}-YFP activation in movie experiments.

(A-D) (JLB130) and ΔepsH (JLB221) were grown in SMM in the mother machine microfluidics device for 240 min before switching the media to SMM with 1 µg/ml lysozyme. (E) Each line corresponds to the fraction of cells with a P_{sigV}-YFP larger than its half maximum at a given time point. WT (JLB130) and ΔepsH (JLB221) were grown in the mother machine in SMM for 240 min before switching to 1 µg/ml lysozyme (n=2).

Data information: For more information on the number of repeats please see Appendix Table S5.

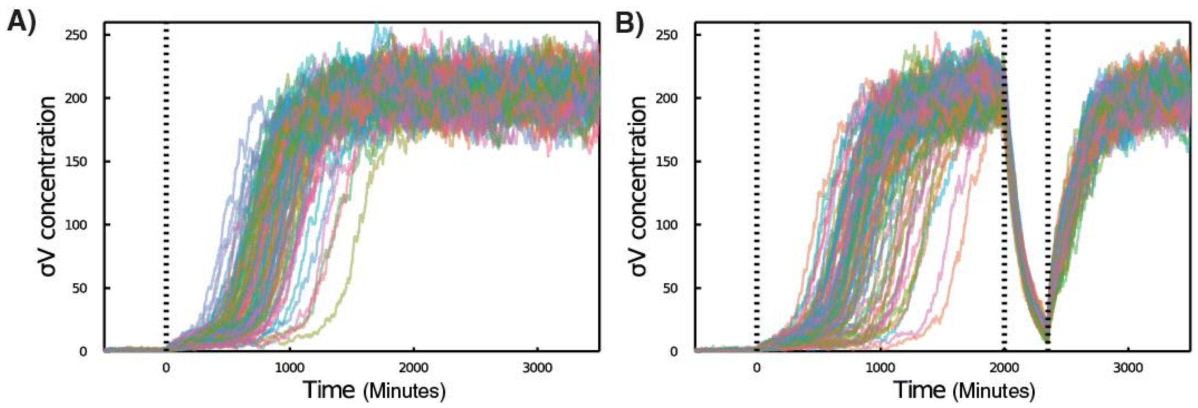

Appendix Figure S33. A continuous, chemical Langevin equation based, SDE model can also capture the system behaviours.

All figures contain 100 trajectories from a chemical Langevin equation based SDE model of the system. The same reactions are modelled, albeit with a slightly modified parameter set, (v_0,v,K,n,kD,kB,kC,k_{deg})=(*0.025 au*·min^{-1} ,*2.0 au*·min^{-1},*50.0 au* , *2*, *5.0* min^{-1}, *100.0 au*·min^{-1}, *0.025*min^{-1}*, 0.01* min^{-}). (A) The CLE based model displays the same heterogeneous activation behaviour as the SSA model (stress added at the black dashed line). (B) It also contains the same memory mechanism (stress is added at the first and third black dashed lines, and removed at the second one).

Data information: For more information on the number of repeats please see Appendix Table S5.

2. Appendix Tables

Appendix Table S1. Strain list

**Strain Name**	**Genotype**	**Source**
PY79	BGSC 1A747	BGSC
JP1/JLB54	PY79 *ppsb*::P_{trpE}-mCh ($Phleo^{R}$)	(Park et al. 2018)
JP2	JP1 *ytvA*::*neo*	(Park et al. 2018)
JLB047	JP2 *sacA*::P_{sigV}-YFP (Cm^{R})	This work
JLB130	JLB047 *haG*::*erm*	This work
JLB154	JLB130 *sigV*::*tet*	This work
JLB156	JLB130 *oatA*::*tet*	This work
JLB191	JLB130 *amyE*::$P_{hyperspank}$-*pbpX* ($Spec^{R}$)	This work
JLB193	JLB130 *amyE*::$P_{hyperspank}$-*rsiV* ($Spec^{R}$)	This work
JLB196	JLB *amyE*::P_{spank}-*rsiV* ($Spec^{R}$)	This work
JLB208	JLB130 *rsiV*::*tet*	This work
JLB209	JLB130 *amyE*:: P_{spank}-*sigV* ($Spec^{R}$)	This work
JLB210	JLB130 *amyE*::P_{sigV}-*sigV* ($Spec^{R}$)	This work
JLB211	JLB130 *amyE*:: P_{spank}-*sigVrsiV* ($Spec^{R}$)	This work
JLB212	JLB130 *amyE*::P_{sigV}-*rsiV* ($Spec^{R}$)	This work
JLB213	JLB130 *amyE*::P_{sigV}-mTurq ($Spec^{R}$)	This work

JLB215	JLB130 *amyE*::$P_{hyperspank}$-*yrhK* ($Spec^R$)	This work
JLB216	JLB130 *amyE*::$P_{hyperspank}$-*sipS* ($Spec^R$)	This work
JLB217	JLB130 *amyE*::$P_{hyperspank}$-*rasP* ($Spec^R$)	This work
JLB218	JLB130 *amyE*::$P_{hyperspank}$-*oatA* ($Spec^R$)	This work
JLB219	JLB130 *amyE*::$P_{hyperspank}$-*sigV* ($Spec^R$)	This work
JLB221	JLB130 *epsH*::*tet*	This work
JLB232	JLB130 *sigVrsiVoatAyrhK*::*tet*	This work
JLB236	JLB232 *amyE*:: P_{spank}-*sigVrsiVoatAyrhK* ($Spec^R$)	This work
JLB242	JLB54 *amyE*::P_{spank}-YFP ($Spec^R$)	This work
JLB244	JLB54 *amyE*::$P_{hyperspank}$-YFP ($Spec^R$)	This work

Appendix Table S2. Model parameters

Parameter values for the model were set as follows.

Parameter	Description	Value
v_0	Leakage of the operon	0.1 Molecules•min^{-1}
v	Maximal operon activity	2.5 Molecules•min^{-1}
K	Apparent dissociation constant	60 Molecules
n	Hill coefficient	2
k_D	Dissociation rate	5 min^{-1}
k_B	Binding rate	10 $Molecules^{-1}$ min^{-1}
k_C	Cleavage rate	0.05 min^{-1}
k_{deg}	Dilution/degradation rate	0.01 min^{-1}
L	Lysozyme stress strength	variable

Appendix Table S3. Number of repeats in the main figures

Figure	Biological Repeats (n)	Cells (N)	Strain
1D	2	95	JLB130
1E	2	95	JLB130
1F	1<br>The second repeat is in Appendix Figure S 7	0.5 µg/ml: 45<br>1.0 µg/ml: 58<br>2.0 µg/ml: 62<br>4.0 µg/ml: 53	JLB130
1G	2	0.0 µg/ml Repeat 1: 61<br>0.0 µg/ml Repeat 2: 50<br>0.5 µg/ml Repeat 1: 45<br>0.5 µg/ml Repeat 2: 50<br>1.0 µg/ml Repeat 1: 58<br>1.0 µg/ml Repeat 2: 51<br>2.0 µg/ml Repeat 1: 62<br>2.0 µg/ml Repeat 2: 52<br>4.0 µg/ml Repeat 1: 53<br>4.0 µg/ml Repeat 2: 47	JLB130
2B	No Pre-Stress: n=2<br>Pre-Stress: n=4	Priming Repeat 1: 1068<br>Priming Repeat 2: 773<br>Priming Repeat 3: 930<br>Priming Repeat 4: 2166<br>No Priming Repeat 1: 981<br>No Priming Repeat 2: 1032	JLB130
2C	n=4<br>The shown data is corrected data only	Survivors Repeat 1: 46<br>Perishers Repeat 1: 77<br>Survivors Repeat 2: 30<br>Perishers Repeat 2: 49<br>Survivors Repeat 3: 120	JLB130

		Perishers Repeat 3: 122<br>Survivors Repeat 4: 54<br>Perishers Repeat 4: 66	
3B	n=2	Not applicable	JLB130
3C	n=2	Not applicable	JLB154
3D	n≥3<br><br>Histograms in Appendix Figure S16	WT 0 Lyso & 1 IPTG n: 8<br>WT 0 Lyso & 1 IPTG Repeat 1: 3186<br>WT 0 Lyso & 1 IPTG Repeat 2: 2820<br>WT 0 Lyso & 1 IPTG Repeat 3: 2139<br>WT 0 Lyso & 1 IPTG Repeat 4: 3325<br>WT 0 Lyso & 1 IPTG Repeat 5: 2529<br>WT 0 Lyso & 1 IPTG Repeat 6: 2898<br>WT 0 Lyso & 1 IPTG Repeat 7: 2012<br>WT 0 Lyso & 1 IPTG Repeat 8: 2839<br>WT 1 Lyso & 1 IPTG Repeat 1: 3150<br>WT 1 Lyso & 1 IPTG Repeat 2: 3938<br>WT 1 Lyso & 1 IPTG Repeat 3: 2264<br>WT 1 Lyso & 1 IPTG Repeat 4: 4493<br>WT 1 Lyso & 1 IPTG Repeat 5: 2677<br>WT 1 Lyso & 1 IPTG Repeat 6: 2832<br>WT 1 Lyso & 1 IPTG Repeat 7: 2324<br>WT 1 Lyso & 1 IPTG Repeat 8: 4480<br>sigV 0 Lyso & 1 IPTG Repeat 1: 906<br>sigV 0 Lyso & 1 IPTG Repeat 2: 2122<br>sigV 0 Lyso & 1 IPTG Repeat 3: 1647<br>sigV 0 Lyso & 1 IPTG Repeat 4: 1721<br>sigV 1 Lyso & 1 IPTG Repeat 1: 1963<br>sigV 1 Lyso & 1 IPTG Repeat 2: 2386<br>sigV 1 Lyso & 1 IPTG Repeat 3: 1802<br>sigV 1 Lyso & 1 IPTG Repeat 4: 1669	WT: JLB130<br>*sigV+:* JLB219<br>*rsiV+:* JLB193<br>*oatA*+: JLB218<br>*yrhK*+: JLB215<br>*sipS*+: JLB216<br>*rasP*+: JLB217<br>*pbpX*+: JLB191

		rsiV 0 Lyso & 1 IPTG Repeat 1: 2300<br>rsiV 0 Lyso & 1 IPTG Repeat 2: 2612<br>rsiV 0 Lyso & 1 IPTG Repeat 3: 1856<br>rsiV 1 Lyso & 1 IPTG Repeat 1: 2919<br>rsiV 1 Lyso & 1 IPTG Repeat 2: 2098<br>rsiV 1 Lyso & 1 IPTG Repeat 3: 2397<br>oatA 0 Lyso & 1 IPTG Repeat 1: 3898<br>oatA 0 Lyso & 1 IPTG Repeat 2: 2850<br>oatA 0 Lyso & 1 IPTG Repeat 3: 3743<br>oatA 1 Lyso & 1 IPTG Repeat 1: 2147<br>oatA 1 Lyso & 1 IPTG Repeat 2: 3025<br>oatA 1 Lyso & 1 IPTG Repeat 3: 2449<br>oatA 1 Lyso & 1 IPTG Repeat 4: 4200<br>yrhK 0 Lyso & 1 IPTG Repeat 1: 2107<br>yrhK 0 Lyso & 1 IPTG Repeat 2: 936<br>yrhK 0 Lyso & 1 IPTG Repeat 3: 2528<br>yrhK 1 Lyso & 1 IPTG Repeat 1: 2130<br>yrhK 1 Lyso & 1 IPTG Repeat 2: 2585<br>yrhK 1 Lyso & 1 IPTG Repeat 3: 3071<br>sipS 0 Lyso & 1 IPTG Repeat 1: 2683<br>sipS 0 Lyso & 1 IPTG Repeat 2: 2837<br>sipS 0 Lyso & 1 IPTG Repeat 3: 3008<br>sipS 1 Lyso & 1 IPTG Repeat 1: 914<br>sipS 1 Lyso & 1 IPTG Repeat 2: 2999<br>sipS 1 Lyso & 1 IPTG Repeat 3: 4081<br>rasP 0 Lyso & 1 IPTG Repeat 1: 2133<br>rasP 0 Lyso & 1 IPTG Repeat 2: 2081<br>rasP 0 Lyso & 1 IPTG Repeat 3: 3466<br>rasP 1 Lyso & 1 IPTG Repeat 1: 2741<br>rasP 1 Lyso & 1 IPTG Repeat 2: 3194<br>rasP 1 Lyso & 1 IPTG Repeat 3: 2970<br>pbpX 0 Lyso & 1 IPTG Repeat 1: 6033	

		pbpX 0 Lyso & 1 IPTG Repeat 2: 9011<br>pbpX 0 Lyso & 1 IPTG Repeat 3: 7330<br>pbpX 0 Lyso & 1 IPTG Repeat 4: 2824<br>pbpX 0 Lyso & 1 IPTG Repeat 5: 3238<br>pbpX 0 Lyso & 1 IPTG Repeat 6: 7260<br>pbpX 1 Lyso & 1 IPTG Repeat 1: 6033<br>pbpX 1 Lyso & 1 IPTG Repeat 2: 6873<br>pbpX 1 Lyso & 1 IPTG Repeat 3: 8754<br>pbpX 1 Lyso & 1 IPTG Repeat 4: 3540<br>pbpX 1 Lyso & 1 IPTG Repeat 5: 4884<br>pbpX 1 Lyso & 1 IPTG Repeat 6: 7643	
3E	n≥3<br>Histograms in Appendix Figure S17	WT 0 Lyso Repeat 1: 1228<br>WT 0 Lyso Repeat 2: 1828<br>WT 0 Lyso Repeat 3: 2122<br>WT 0 Lyso Repeat 4: 1648<br>WT 0 Lyso Repeat 5: 1994<br>WT 0 Lyso Repeat 6: 1849<br>WT 0 Lyso Repeat 7: 2776<br>WT 0 Lyso Repeat 8: 2216<br>WT 0 Lyso Repeat 9: 2984<br>WT 1 Lyso Repeat 1: 1450<br>WT 1 Lyso Repeat 2: 1794<br>WT 1 Lyso Repeat 3: 2813<br>WT 1 Lyso Repeat 4: 1052<br>WT 1 Lyso Repeat 5: 1494<br>WT 1 Lyso Repeat 6: 2190<br>WT 1 Lyso Repeat 7: 2923<br>WT 1 Lyso Repeat 8: 2997<br>WT 1 Lyso Repeat 9: 6731<br>ΔsigV 0 Lyso Repeat 1: 4531<br>ΔsigV 0 Lyso Repeat 2: 1349<br>ΔsigV 0 Lyso Repeat 3: 1213	WT: JLB130<br>ΔsigV:<br>JLB154<br>ΔrsiV:<br>JLB208<br>ΔoatA:<br>JLB156

		ΔsigV 1 Lyso Repeat 1: 5163<br>ΔsigV 1 Lyso Repeat 2: 1552<br>ΔsigV 1 Lyso Repeat 3: 3066<br>ΔrsiV 0 Lyso Repeat 1: 2160<br>ΔrsiV 0 Lyso Repeat 2: 2606<br>ΔrsiV 0 Lyso Repeat 3: 2807<br>ΔrsiV 1 Lyso Repeat 1: 1922<br>ΔrsiV 1 Lyso Repeat 2: 2308<br>ΔrsiV 1 Lyso Repeat 3: 2311<br>ΔoatA 0 Lyso Repeat 1: 1339<br>ΔoatA 0 Lyso Repeat 2: 888<br>ΔoatA 0 Lyso Repeat 3: 3551<br>ΔoatA 1 Lyso Repeat 1: 1208<br>ΔoatA 1 Lyso Repeat 2: 800<br>ΔoatA 1 Lyso Repeat 3: 3569	
4A	Not applicable	100	Not applicable
4B	Not applicable	99	Not applicable
4C	Not applicable	99	Not applicable
5B	1000 bootstraps	99	Not applicable
5C	n=3	WT Repeat 1: 499<br>WT Repeat 2: 624<br>WT Repeat 3: 365<br>2x sigV Repeat 1: 454<br>2x sigV Repeat 2: 443<br>2x sigV Repeat 3: 400<br>2x rsiV Repeat 1: 510<br>2x rsiV Repeat 2: 674<br>2x rsiV Repeat 3: 502<br>2x sigVrsiV Repeat 1: 434	WT: JLB130<br>2xsigV: JLB210<br>2xrsiV: JLB212<br>2xsigVrsiV: JLB211

		2x sigVrsiV Repeat 2: 475<br>2x sigVrsiV Repeat 3: 342	
6A	Not applicable	99	Not applicable
6B	Not applicable	99	Not applicable
6C	Not applicable	300 au break: 500<br>450 au break: 500<br>600 au break: 500	Not applicable
6D	1 the repeat is in Appendix Figure S24	48	JLB221
6E	1 the repeat is in Appendix Figure S24	54	JLB221
6F	1 the repeat is in Appendix Figure S24	6h no stress: 48<br>12h no stress: 54<br>Control: 56	JLB221

Appendix Table S4. Number of repeats in the extended figures

Figure	Biological Repeats (n)	Cells (N)	Strain
EV1	2 all repeats are shown in the figure	A) 0.5 µg/ml Repeat 1: 45<br>B) 0.5 µg/ml Repeat 2: 50<br>C) 1.0 µg/ml Repeat 1: 58<br>D) 1.0 µg/ml Repeat 2: 51<br>E) 2.0 µg/ml Repeat 1: 62<br>F) 2.0 µg/ml Repeat 2: 52<br>G) 4.0 µg/ml Repeat 1: 53<br>H) 4.0 µg/ml Repeat 2: 47	JLB130
EV2	2	A) 0.5 µg/ml:<br>Red: 11; Blue: 84<br>B) 1.0 µg/ml:<br>Red: 11; Blue: 98<br>C) 2.0 µg/ml:<br>Red: 15; Blue: 97<br>D) 4.0 µg/ml: 86<br>Red: 12; Blue: 74	JLB130
EV3	n=3	WT 0 Lyso & 1 IPTG Repeat 1: 2820<br>WT 0 Lyso & 1 IPTG Repeat 2: 2529<br>WT 0 Lyso & 1 IPTG Repeat 3: 2012<br>WT 1 Lyso & 1 IPTG Repeat 1: 3938<br>WT 1 Lyso & 1 IPTG Repeat 2: 2677<br>WT 1 Lyso & 1 IPTG Repeat 3: 2324<br>oatA+ 1 Lyso & 1 IPTG Repeat 1: 2147<br>oatA+ 1 Lyso & 1 IPTG Repeat 2: 2449<br>oatA+ 1 Lyso & 1 IPTG Repeat 3: 4200<br>oatA+ 20 Lyso & 1 IPTG Repeat 1: 2730<br>oatA+ 20 Lyso & 1 IPTG Repeat 2: 2501<br>oatA+ 20 Lyso & 1 IPTG Repeat 3: 2906	WT: JLB130<br>OatA+: JLB218

EV4	Not applicable	100	Not applicable
EV5	n≥3<br>See Appendix Figure 24 for histograms	blank 0L 0I Repeat 1: 4123<br>blank 0L 0I Repeat 2: 4660<br>blank 0L 0I Repeat 3: 2215<br>blank 0L 0I Repeat 4: 3969<br>blank 1L 0I Repeat 1: 4174<br>blank 1L 0I Repeat 2: 3275<br>blank 1L 0I Repeat 3: 4652<br>blank 1L 0I Repeat 4: 1652<br>blank 0L 1I Repeat 1: 4256<br>blank 0L 1I Repeat 2: 4126<br>blank 0L 1I Repeat 3: 3141<br>blank 0L 1I Repeat 4: 3541<br>blank 1L 1I Repeat 1: 2963<br>blank 1L 1I Repeat 2: 5546<br>blank 1L 1I Repeat 3: 3230<br>blank 1L 1I Repeat 4: 2850<br>WT 0L 0I Repeat 1: 2918<br>WT 0L 0I Repeat 2: 3475<br>WT 0L 0I Repeat 3: 3985<br>WT 0L 0I Repeat 4: 2486<br>WT 1L 0I Repeat 1: 2116<br>WT 1L 0I Repeat 2: 3636<br>WT 1L 0I Repeat 3: 5481<br>WT 1L 0I Repeat 4: 2826<br>WT 0L 1I Repeat 1: 4499<br>WT 0L 1I Repeat 2: 3543<br>WT 0L 1I Repeat 3: 5179<br>WT 0L 1I Repeat 4: 2532<br>WT 1L 1I Repeat 1: 4229<br>WT 1L 1I Repeat 2: 3130<br>WT 1L 1I Repeat 3: 4101	JLB236

		WT 1L 1I Repeat 4: 2397<br>operon 0L 0I Repeat 1: 5108<br>operon 0L 0I Repeat 2: 3669<br>operon 0L 0I Repeat 3: 4419<br>operon 0L 0I Repeat 4: 1683<br>operon 1L 0I Repeat 1: 5509<br>operon 1L 0I Repeat 2: 4454<br>operon 1L 0I Repeat 3: 4911<br>operon 1L 0I Repeat 4: 2965<br>operon 0L 1I Repeat 1: 4608<br>operon 0L 1I Repeat 2: 5033<br>operon 0L 1I Repeat 3: 4945<br>operon 0L 1I Repeat 4: 3338<br>operon 1L 1I Repeat 1: 7688<br>operon 1L 1I Repeat 2: 6133<br>operon 1L 1I Repeat 3: 6064<br>operon 1L 1I Repeat 4: 3941<br>operon 0L 2I Repeat 1: 6041<br>operon 0L 2I Repeat 2: 4315<br>operon 0L 2I Repeat 3: 3574<br>operon 0L 2I Repeat 4: 434<br>operon 1L 2I Repeat 1: 4557<br>operon 1L 2I Repeat 2: 4851<br>operon 1L 2I Repeat 3: 4235<br>operon 1L 2I Repeat 4: 2615<br>operon 0L 5I Repeat 1: 4921<br>operon 0L 5I Repeat 2: 6273<br>operon 0L 5I Repeat 3: 5774<br>operon 0L 5I Repeat 4: 2824<br>operon 1L 5I Repeat 1: 5825<br>operon 1L 5I Repeat 2: 5089<br>operon 1L 5I Repeat 3: 5178	

		operon 1L 5I Repeat 4: 2912<br>operon 0L 10I Repeat 1: 4359<br>operon 0L 10I Repeat 2: 5366<br>operon 0L 10I Repeat 3: 4622<br>operon 0L 10I Repeat 4: 3813<br>operon 1L 10I Repeat 1: 5343<br>operon 1L 10I Repeat 2: 4772<br>operon 1L 10I Repeat 3: 3304<br>operon 1L 10I Repeat 4: 4035<br>operon 0L 20I Repeat 1: 6554<br>operon 0L 20I Repeat 2: 5535<br>operon 0L 20I Repeat 3: 4553<br>operon 0L 20I Repeat 4: 3381<br>operon 1L 20I Repeat 1: 4322<br>operon 1L 20I Repeat 2: 6707<br>operon 1L 20I Repeat 3: 5988<br>operon 1L 20I Repeat 4: 4072<br>operon 0L 50I Repeat 1: 4989<br>operon 0L 50I Repeat 2: 6820<br>operon 0L 50I Repeat 3: 3615<br>operon 0L 50I Repeat 4: 4361<br>operon 1L 50I Repeat 1: 4995<br>operon 1L 50I Repeat 2: 3282<br>operon 1L 50I Repeat 3: 3921<br>operon 1L 50I Repeat 4: 3417<br>operon 0L 100I Repeat 1: 859<br>operon 0L 100I Repeat 2: 4700<br>operon 0L 100I Repeat 3: 5033<br>operon 0L 100I Repeat 4: 3888<br>operon 1L 100I Repeat 1: 5455<br>operon 1L 100I Repeat 2: 6740<br>operon 1L 100I Repeat 3: 1337	

		operon 1L 100I Repeat 4: 4655<br>operon 0L 200I Repeat 1: 3927<br>operon 0L 200I Repeat 2: 4281<br>operon 0L 200I Repeat 3: 3534<br>operon 1L 200I Repeat 1: 3649<br>operon 1L 200I Repeat 2: 5822<br>operon 1L 200I Repeat 3: 3279<br>operon 0L 500I Repeat 1: 6344<br>operon 0L 500I Repeat 2: 5218<br>operon 0L 500I Repeat 3: 2856<br>operon 1L 500I Repeat 1: 5515<br>operon 1L 500I Repeat 2: 4911<br>operon 1L 500I Repeat 3: 3818<br>operon 0L 1000I Repeat 1: 2616<br>operon 0L 1000I Repeat 2: 5865<br>operon 0L 1000I Repeat 3: 4822<br>operon 0L 1000I Repeat 4: 4976<br>operon 1L 1000I Repeat 1: 4374<br>operon 1L 1000I Repeat 2: 5051<br>operon 1L 1000I Repeat 3: 2244<br>operon 1L 1000I Repeat 4: 4713	

Appendix Table S5. Number of repeats in the appendix figures

Figure	Biological Repeats (n)	Cells (N)	Strain
S1	2<br>All repeats are shown in the figure	A) 0.5 µg/ml Repeat 1: 45<br>B) 0.5 µg/ml Repeat 2: 50<br>C) 1.0 µg/ml Repeat 1: 58<br>D) 1.0 µg/ml Repeat 2: 51<br>E) 2.0 µg/ml Repeat 1: 62<br>F) 2.0 µg/ml Repeat 2: 52<br>G) 4.0 µg/ml Repeat 1: 53<br>H) 4.0 µg/ml Repeat 2: 47	JLB130
S2	2<br>All repeats are shown in the figure	A) 0.5 µg/ml Repeat 1: 45<br>B) 0.5 µg/ml Repeat 2: 50<br>C) 1.0 µg/ml Repeat 1: 58<br>D) 1.0 µg/ml Repeat 2: 51<br>E) 2.0 µg/ml Repeat 1: 62<br>F) 2.0 µg/ml Repeat 2: 52<br>G) 4.0 µg/ml Repeat 1: 53<br>H) 4.0 µg/ml Repeat 2: 47	JLB130
S3	2	A) 0.5 µg/ml: 95<br>B) 1.0 µg/ml: 109<br>C) 2.0 µg/ml: 114<br>D) 4.0 µg/ml: 100	JLB130
S4	Movie data (red lines): 2<br>Snap data (black lines): 8	Movie Data Repeat 1: 766<br>Movie Data Repeat 2: 744<br>Snap Data Repeat 1: 3144<br>Snap Data Repeat 2: 3443<br>Snap Data Repeat 3: 2901<br>Snap Data Repeat 4: 2667<br>Snap Data Repeat 5: 3736<br>Snap Data Repeat 6: 3090<br>Snap Data Repeat 7: 2687	JLB130

		Snap Data Repeat 8: 4239	
S5	3	Cell at the end of the movie:<br>A) 0.0 µg/ml: 1003<br>B) 0.5 µg/ml: 626<br>C) 1.0 µg/ml: 573<br>D) 4.0 µg/ml: 825	JLB47
S6	3	3238	JLB213
S7	2<br>All repeats are shown in the figure	A and B)<br>0.5 µg/ml Repeat 1: 45<br>0.5 µg/ml Repeat 2: 50<br>1.0 µg/ml Repeat 1: 58<br>1.0 µg/ml Repeat 2: 51<br>2.0 µg/ml Repeat 1: 62<br>2.0 µg/ml Repeat 2: 52<br>4.0 µg/ml Repeat 1: 53<br>4.0 µg/ml Repeat 2: 47	JLB130
S8	1<br>The second repeat is in Figure 1E	0.5 µg/ml: 50<br>1.0 µg/ml: 51<br>2.0 µg/ml: 52<br>4.0 µg/ml: 47	JLB130
S9	2<br>All repeats are shown in the figure	A) 0.5 µg/ml Repeat 1: 45<br>B) 0.5 µg/ml Repeat 2: 50<br>C) 1.0 µg/ml Repeat 1: 58<br>D) 1.0 µg/ml Repeat 2: 51<br>E) 2.0 µg/ml Repeat 1: 61<br>F) 2.0 µg/ml Repeat 2: 51<br>G) 4.0 µg/ml Repeat 1: 49<br>H) 4.0 µg/ml Repeat 2: 37	JLB130
S10	2:<br>Two red lines:	Red lines:	JLB130

	All Cells<br>Two blue lines: Overshooters removed	Repeat 1: 53<br>Repeat 2: 47<br>Blue lines:<br>Repeat 1: 49<br>Repeat 2: 37	
S11	2	A) 0.5 µg/ml: 72<br>B) 1.0 µg/ml: 94<br>C) 2.0 µg/ml: 72<br>D) 4.0 µg/ml: 53	JLB130
S12	2	A) 0.5 µg/ml: 95<br>B) 1.0 µg/ml: 109<br>C) 2.0 µg/ml: 112<br>D) 4.0 µg/ml: 86	JLB130
S13	2	YFP & RFP Repeat 1: 61<br>YFP & RFP Repeat 2: 50	JLB130
S14	2	A) 0.5 µg/ml:<br>Red: 11; Blue: 84<br>B) 1.0 µg/ml:<br>Red: 11; Blue: 98<br>C) 2.0 µg/ml:<br>Red: 15; Blue: 97<br>D) 4.0 µg/ml: 86<br>Red: 12; Blue: 74	JLB130
S15	A) n=2<br>B) n=4	A) N=113<br>B) Survivors: 250<br>Perishers: 326	JLB130
S16	WT 0 Lyso & 0 IPTG n: 8<br>WT 1 Lyso & 0 IPTG n: 8<br>WT 0 Lyso & 1 IPTG n: 8<br>WT 1 Lyso & 1 IPTG n: 8<br>sigV+ 0 Lyso & 0 IPTG n: 4<br>sigV+ 1 Lyso & 0 IPTG n: 4	WT 0 Lyso & 0 IPTG Repeat 1: 3019<br>WT 0 Lyso & 0 IPTG Repeat 2: 2720<br>WT 0 Lyso & 0 IPTG Repeat 3: 2721<br>WT 0 Lyso & 0 IPTG Repeat 4: 3690<br>WT 0 Lyso & 0 IPTG Repeat 5: 2142<br>WT 0 Lyso & 0 IPTG Repeat 6: 4295	WT: JLB130<br>*sigV+:* JLB219<br>*RsiV+:* JLB193<br>*oatA*+: JLB218<br>*yrhK*+: JLB215<br>*sipS*+: JLB216

	sigV+ 0 Lyso & 1 IPTG n: 4<br>sigV+ 1 Lyso & 1 IPTG n: 4<br>rsiV+ 0 Lyso & 0 IPTG n: 3<br>rsiV+ 1 Lyso & 0 IPTG n: 3<br>rsiV+ 0 Lyso & 1 IPTG n: 3<br>rsiV+ 1 Lyso & 1 IPTG n: 3<br>oatA+ 0 Lyso & 0 IPTG n: 3<br>oatA+ 1 Lyso & 0 IPTG n: 3<br>oatA+ 0 Lyso & 1 IPTG n: 3<br>oatA+ 1 Lyso & 1 IPTG n: 3<br>yrhK+ 0 Lyso & 0 IPTG n: 3<br>yrhK+ 1 Lyso & 0 IPTG n: 3<br>yrhK+ 0 Lyso & 1 IPTG n: 3<br>yrhK+ 1 Lyso & 1 IPTG n: 3<br>sipS+ 0 Lyso & 0 IPTG n: 3<br>sipS+ 1 Lyso & 0 IPTG n: 3<br>sipS+ 0 Lyso & 1 IPTG n: 3<br>sipS+ 1 Lyso & 1 IPTG n: 3<br>rasP+ 0 Lyso & 0 IPTG n: 3<br>rasP+ 1 Lyso & 0 IPTG n: 3<br>rasP+ 0 Lyso & 1 IPTG n: 3<br>rasP+ 1 Lyso & 1 IPTG n: 3<br>pbpX+ 0 Lyso & 0 IPTG n: 6<br>pbpX + 1 Lyso & 0 IPTG n: 6<br>pbpX + 0 Lyso & 1 IPTG n: 6<br>pbpX + 1 Lyso & 1 IPTG n: 6	WT 0 Lyso & 0 IPTG Repeat 7: 2553<br>WT 0 Lyso & 0 IPTG Repeat 8: 4295<br>WT 1 Lyso & 0 IPTG Repeat 1: 3072<br>WT 1 Lyso & 0 IPTG Repeat 2: 3012<br>WT 1 Lyso & 0 IPTG Repeat 3: 2752<br>WT 1 Lyso & 0 IPTG Repeat 4: 2543<br>WT 1 Lyso & 0 IPTG Repeat 5: 3710<br>WT 1 Lyso & 0 IPTG Repeat 6: 3077<br>WT 1 Lyso & 0 IPTG Repeat 7: 2647<br>WT 1 Lyso & 0 IPTG Repeat 8: 4182<br>WT 0 Lyso & 1 IPTG Repeat 1: 3186<br>WT 0 Lyso & 1 IPTG Repeat 2: 2820<br>WT 0 Lyso & 1 IPTG Repeat 3: 2139<br>WT 0 Lyso & 1 IPTG Repeat 4: 3325<br>WT 0 Lyso & 1 IPTG Repeat 5: 2529<br>WT 0 Lyso & 1 IPTG Repeat 6: 2898<br>WT 0 Lyso & 1 IPTG Repeat 7: 2012<br>WT 0 Lyso & 1 IPTG Repeat 8: 2839<br>WT 1 Lyso & 1 IPTG Repeat 1: 3150<br>WT 1 Lyso & 1 IPTG Repeat 2: 3938<br>WT 1 Lyso & 1 IPTG Repeat 3: 2264<br>WT 1 Lyso & 1 IPTG Repeat 4: 4493<br>WT 1 Lyso & 1 IPTG Repeat 5: 2677<br>WT 1 Lyso & 1 IPTG Repeat 6: 2832<br>WT 1 Lyso & 1 IPTG Repeat 7: 2324<br>WT 1 Lyso & 1 IPTG Repeat 8: 4480<br>sigV+ 0 Lyso & 0 IPTG Repeat 1: 2742<br>sigV+ 0 Lyso & 0 IPTG Repeat 2: 3401<br>sigV+ 0 Lyso & 0 IPTG Repeat 3: 4468<br>sigV+ 0 Lyso & 0 IPTG Repeat 4: 4637<br>sigV+ 1 Lyso & 0 IPTG Repeat 1: 3362<br>sigV+ 1 Lyso & 0 IPTG Repeat 2: 3510	*rasP+*: JLB217<br>*pbpX+*: JLB191

		sigV+ 1 Lyso & 0 IPTG Repeat 3: 3515<br>sigV+ 1 Lyso & 0 IPTG Repeat 4: 2947<br>sigV+ 0 Lyso & 1 IPTG Repeat 1: 906<br>sigV+ 0 Lyso & 1 IPTG Repeat 2: 2122<br>sigV+ 0 Lyso & 1 IPTG Repeat 3: 1647<br>sigV+ 0 Lyso & 1 IPTG Repeat 4: 1721<br>sigV+ 1 Lyso & 1 IPTG Repeat 1: 1963<br>sigV+ 1 Lyso & 1 IPTG Repeat 2: 2386<br>sigV+ 1 Lyso & 1 IPTG Repeat 3: 1802<br>sigV+ 1 Lyso & 1 IPTG Repeat 4: 1669<br>rsiV+ 0 Lyso & 0 IPTG Repeat 1: 3264<br>rsiV+ 0 Lyso & 0 IPTG Repeat 2: 2319<br>rsiV+ 0 Lyso & 0 IPTG Repeat 3: 1399<br>rsiV+ 1 Lyso & 0 IPTG Repeat 1: 2734<br>rsiV+ 1 Lyso & 0 IPTG Repeat 2: 3317<br>rsiV+ 1 Lyso & 0 IPTG Repeat 3: 2148<br>rsiV+ 0 Lyso & 1 IPTG Repeat 1: 2300<br>rsiV+ 0 Lyso & 1 IPTG Repeat 2: 2612<br>rsiV+ 0 Lyso & 1 IPTG Repeat 3: 1856<br>rsiV+ 1 Lyso & 1 IPTG Repeat 1: 2919<br>rsiV+ 1 Lyso & 1 IPTG Repeat 2: 2098<br>rsiV+ 1 Lyso & 1 IPTG Repeat 3: 2397<br>oatA+ 0 Lyso & 0 IPTG Repeat 1: 3763<br>oatA+ 0 Lyso & 0 IPTG Repeat 2: 3170<br>oatA+ 0 Lyso & 0 IPTG Repeat 3: 3342<br>oatA+ 1 Lyso & 0 IPTG Repeat 1: 2783<br>oatA+ 1 Lyso & 0 IPTG Repeat 2: 3216<br>oatA+ 1 Lyso & 0 IPTG Repeat 3: 4297<br>oatA+ 0 Lyso & 1 IPTG Repeat 1: 3898<br>oatA+ 0 Lyso & 1 IPTG Repeat 2: 2850<br>oatA+ 0 Lyso & 1 IPTG Repeat 3: 3743<br>oatA+ 1 Lyso & 1 IPTG Repeat 1: 2147	

		oatA+ 1 Lyso & 1 IPTG Repeat 2: 2449<br>oatA+ 1 Lyso & 1 IPTG Repeat 3: 4200<br>yrhK+ 0 Lyso & 0 IPTG Repeat 1: 3088<br>yrhK+ 0 Lyso & 0 IPTG Repeat 2: 3295<br>yrhK+ 0 Lyso & 0 IPTG Repeat 3: 3282<br>yrhK+ 1 Lyso & 0 IPTG Repeat 1: 2116<br>yrhK+ 1 Lyso & 0 IPTG Repeat 2: 3285<br>yrhK+ 1 Lyso & 0 IPTG Repeat 3: 2936<br>yrhK+ 0 Lyso & 1 IPTG Repeat 1: 2107<br>yrhK+ 0 Lyso & 1 IPTG Repeat 2: 936<br>yrhK+ 0 Lyso & 1 IPTG Repeat 3: 2528<br>yrhK+ 1 Lyso & 1 IPTG Repeat 1: 2130<br>yrhK+ 1 Lyso & 1 IPTG Repeat 2: 2585<br>yrhK+ 1 Lyso & 1 IPTG Repeat 3: 3071<br>sipS+ 0 Lyso & 0 IPTG Repeat 1: 2221<br>sipS+ 0 Lyso & 0 IPTG Repeat 2: 3401<br>sipS+ 0 Lyso & 0 IPTG Repeat 3: 3940<br>sipS+ 1 Lyso & 0 IPTG Repeat 1: 2478<br>sipS+ 1 Lyso & 0 IPTG Repeat 2: 2362<br>sipS+ 1 Lyso & 0 IPTG Repeat 3: 3683<br>sipS+ 0 Lyso & 1 IPTG Repeat 1: 2683<br>sipS+ 0 Lyso & 1 IPTG Repeat 2: 2837<br>sipS+ 0 Lyso & 1 IPTG Repeat 3: 3008<br>sipS+ 1 Lyso & 1 IPTG Repeat 1: 914<br>sipS+ 1 Lyso & 1 IPTG Repeat 2: 2999<br>sipS+ 1 Lyso & 1 IPTG Repeat 3: 4081<br>rasP+ 0 Lyso & 0 IPTG Repeat 1: 3499<br>rasP+ 0 Lyso & 0 IPTG Repeat 2: 2561<br>rasP+ 0 Lyso & 0 IPTG Repeat 3: 3482<br>rasP+ 1 Lyso & 0 IPTG Repeat 1: 2645<br>rasP+ 1 Lyso & 0 IPTG Repeat 2: 1607<br>rasP+ 1 Lyso & 0 IPTG Repeat 3: 3626	

		rasP+ 0 Lyso & 1 IPTG Repeat 1: 2133<br>rasP+ 0 Lyso & 1 IPTG Repeat 2: 2081<br>rasP+ 0 Lyso & 1 IPTG Repeat 3: 3466<br>rasP+ 1 Lyso & 1 IPTG Repeat 1: 2741<br>rasP+ 1 Lyso & 1 IPTG Repeat 2: 3194<br>rasP+ 1 Lyso & 1 IPTG Repeat 3: 2970<br>pbpX+ 0 Lyso & 0 IPTG Repeat 1: 6037<br>pbpX+ 0 Lyso & 0 IPTG Repeat 2: 7946<br>pbpX+ 0 Lyso & 0 IPTG Repeat 3: 9833<br>pbpX+ 0 Lyso & 0 IPTG Repeat 4: 4670<br>pbpX+ 0 Lyso & 0 IPTG Repeat 5: 7333<br>pbpX+ 0 Lyso & 0 IPTG Repeat 6: 10043<br>pbpX+ 1 Lyso & 0 IPTG Repeat 1: 8588<br>pbpX+ 1 Lyso & 0 IPTG Repeat 2: 7280<br>pbpX+ 1 Lyso & 0 IPTG Repeat 3: 7795<br>pbpX+ 1 Lyso & 0 IPTG Repeat 4: 9182<br>pbpX+ 1 Lyso & 0 IPTG Repeat 5: 12115<br>pbpX+ 1 Lyso & 0 IPTG Repeat 6: 6034<br>pbpX+ 0 Lyso & 1 IPTG Repeat 1: 6033<br>pbpX+ 0 Lyso & 1 IPTG Repeat 2: 9011<br>pbpX+ 0 Lyso & 1 IPTG Repeat 3: 7330<br>pbpX+ 0 Lyso & 1 IPTG Repeat 4: 2824<br>pbpX+ 0 Lyso & 1 IPTG Repeat 5: 3238<br>pbpX+ 0 Lyso & 1 IPTG Repeat 6: 7260<br>pbpX+ 1 Lyso & 1 IPTG Repeat 1: 6033<br>pbpX+ 1 Lyso & 1 IPTG Repeat 2: 6873<br>pbpX+ 1 Lyso & 1 IPTG Repeat 3: 8754<br>pbpX+ 1 Lyso & 1 IPTG Repeat 4: 3540<br>pbpX+ 1 Lyso & 1 IPTG Repeat 5: 4884<br>pbpX+ 1 Lyso & 1 IPTG Repeat 6: 7643	

S17	WT 0 Lyso n: 9<br>WT 1 Lyso n: 9<br>ΔsigV 0 Lyso n: 3<br>ΔsigV 1 Lyso n: 3<br>ΔrsiV 0 Lyso n: 3<br>ΔrsiV 1 Lyso n: 3<br>ΔoatA 0 Lyso n: 3<br>ΔoatA 1 Lyso n: 3	WT 0 Lyso Repeat 1: 1228<br>WT 0 Lyso Repeat 2: 1828<br>WT 0 Lyso Repeat 3: 2122<br>WT 0 Lyso Repeat 4: 1648<br>WT 0 Lyso Repeat 5: 1994<br>WT 0 Lyso Repeat 6: 1849<br>WT 0 Lyso Repeat 7: 2776<br>WT 0 Lyso Repeat 8: 2216<br>WT 0 Lyso Repeat 9: 2984<br>WT 1 Lyso Repeat 1: 1450<br>WT 1 Lyso Repeat 2: 1794<br>WT 1 Lyso Repeat 3: 2813<br>WT 1 Lyso Repeat 4: 1052<br>WT 1 Lyso Repeat 5: 1494<br>WT 1 Lyso Repeat 6: 2190<br>WT 1 Lyso Repeat 7: 2923<br>WT 1 Lyso Repeat 8: 2997<br>WT 1 Lyso Repeat 9: 6731<br>ΔsigV 0 Lyso Repeat 1: 4531<br>ΔsigV 0 Lyso Repeat 2: 1349<br>ΔsigV 0 Lyso Repeat 3: 1213<br>ΔsigV 1 Lyso Repeat 1: 5163<br>ΔsigV 1 Lyso Repeat 2: 1552<br>ΔsigV 1 Lyso Repeat 3: 3066<br>ΔrsiV 0 Lyso Repeat 1: 2160<br>ΔrsiV 0 Lyso Repeat 2: 2606<br>ΔrsiV 0 Lyso Repeat 3: 2807<br>ΔrsiV 1 Lyso Repeat 1: 1922<br>ΔrsiV 1 Lyso Repeat 2: 2308<br>ΔrsiV 1 Lyso Repeat 3: 2311<br>ΔoatA 0 Lyso Repeat 1: 1339<br>ΔoatA 0 Lyso Repeat 2: 888	WT:<br>JLB130<br>ΔsigV:<br>JLB154<br>ΔrsiV:<br>JLB208<br>ΔoatA:<br>JLB156

		ΔoatA 0 Lyso Repeat 3: 3551<br>ΔoatA 1 Lyso Repeat 1: 1208<br>ΔoatA 1 Lyso Repeat 2: 800<br>ΔoatA 1 Lyso Repeat 3: 3569	
S18	A)<br>blank n: 4<br>Phs-YFP n: 3<br>Ps-YFP n: 3<br><br>B)<br>blank 0L n: 4<br>WT 0L n: 4<br>Ps-sigV 0L n: 3<br>Ps-rsiV 0L n: 3<br>Phs-sigV 0L n: 4<br>Phs-rsiV 0L n: 3<br><br>C)<br>blank 1L n: 4<br>WT 1L n: 4<br>Ps-sigV 1L n: 3<br>Ps-rsiV 1L n: 3<br>Phs-sigV 1L n: 4<br>Phs-rsiV 1L n: 3	A)<br>blank Repeat 1: 4225<br>blank Repeat 2: 6793<br>blank Repeat 3: 2219<br>blank Repeat 4: 3975<br>Phs-YFP Repeat 1: 8219<br>Phs-YFP Repeat 2: 6563<br>Phs-YFP Repeat 3: 5210<br>Ps-YFP Repeat 1: 2997<br>Ps-YFP Repeat 2: 3651<br>Ps-YFP Repeat 3: 3523<br><br>B)<br>blank 0L Repeat 1: 4123<br>blank 0L Repeat 2: 6583<br>blank 0L Repeat 3: 2215<br>blank 0L Repeat 4: 3969<br>WT 0L Repeat 1: 1692<br>WT 0L Repeat 2: 1312<br>WT 0L Repeat 3: 2337<br>WT 0L Repeat 4: 3249<br>Ps-sigV 0L Repeat 1: 1781<br>Ps-sigV 0L Repeat 2: 3566<br>Ps-sigV 0L Repeat 3: 3989<br>Ps-rsiV 0L Repeat 1: 871<br>Ps-rsiV 0L Repeat 2: 1188<br>Ps-rsiV 0L Repeat 3: 3628<br>Phs-sigV 0L Repeat 1: 2742	A)<br>JLB54<br>JLB242<br>JLB244<br><br>B&C)<br>Blank: JLB54,<br>WT: JLB130<br>P_{spank}-sigV: JLB209 P_{spank}-rsiV: JLB196<br>$P_{hyperspank}$-sigV: JLB219<br>$P_{hyperspank}$-rsiV: JLB193

		Phs-sigV 0L Repeat 2: 3401<br>Phs-sigV 0L Repeat 3: 4468<br>Phs-sigV 0L Repeat 4: 4637<br>Phs-rsiV 0L Repeat 1: 3264<br>Phs-rsiV 0L Repeat 2: 2319<br>Phs-rsiV 0L Repeat 3: 1399<br><br>C)<br>blank 1L Repeat 1: 4174<br>blank 1L Repeat 2: 3263<br>blank 1L Repeat 3: 4652<br>blank 1L Repeat 4: 1652<br>WT 1L Repeat 1: 1886<br>WT 1L Repeat 2: 1627<br>WT 1L Repeat 3: 3380<br>WT 1L Repeat 4: 3266<br>Ps-sigV 1L Repeat 1: 1523<br>Ps-sigV 1L Repeat 2: 2135<br>Ps-sigV 1L Repeat 3: 3975<br>Ps-rsiV 1L Repeat 1: 2486<br>Ps-rsiV 1L Repeat 2: 617<br>Ps-rsiV 1L Repeat 3: 2805<br>Phs-sigV 1L Repeat 1: 3362<br>Phs-sigV 1L Repeat 2: 3510<br>Phs-sigV 1L Repeat 3: 3515<br>Phs-sigV 1L Repeat 4: 2947<br>Phs-rsiV 1L Repeat 1: 2734<br>Phs-rsiV 1L Repeat 2: 3317<br>Phs-rsiV 1L Repeat 3: 2148	
S19	blank 0L n: 4<br>blank 1L n: 4<br>WT 0L n: 4	blank 0L Repeat 1: 4123<br>blank 0L Repeat 2: 6583<br>blank 0L Repeat 3: 2215	Blank: JLB54, WT: JLB130 P_{spank}-sigV: JLB209

	WT 1L n: 4<br>Ps-sigV 0L n: 3<br>Ps-sigV 1L n: 3<br>Ps-rsiV 0L n: 3<br>Ps-rsiV 1L n: 3<br>Phs-sigV 0L n: 4<br>Phs-sigV 1L n: 4<br>Phs-rsiV 0L n: 3<br>Phs-rsiV 1L n: 3	blank 0L Repeat 4: 3969<br>blank 1L Repeat 1: 4174<br>blank 1L Repeat 2: 3263<br>blank 1L Repeat 3: 4652<br>blank 1L Repeat 4: 1652<br>WT 0L Repeat 1: 1692<br>WT 0L Repeat 2: 1312<br>WT 0L Repeat 3: 2337<br>WT 0L Repeat 4: 3249<br>WT 1L Repeat 1: 1886<br>WT 1L Repeat 2: 1627<br>WT 1L Repeat 3: 3380<br>WT 1L Repeat 4: 3266<br>Ps-sigV 0L Repeat 1: 1781<br>Ps-sigV 0L Repeat 2: 3566<br>Ps-sigV 0L Repeat 3: 3989<br>Ps-sigV 1L Repeat 1: 1523<br>Ps-sigV 1L Repeat 2: 2135<br>Ps-sigV 1L Repeat 3: 3975<br>Ps-rsiV 0L Repeat 1: 871<br>Ps-rsiV 0L Repeat 2: 1188<br>Ps-rsiV 0L Repeat 3: 3628<br>Ps-rsiV 1L Repeat 1: 2486<br>Ps-rsiV 1L Repeat 2: 617<br>Ps-rsiV 1L Repeat 3: 2805<br>Phs-sigV 0L Repeat 1: 2742<br>Phs-sigV 0L Repeat 2: 3401<br>Phs-sigV 0L Repeat 3: 4468<br>Phs-sigV 0L Repeat 4: 4637<br>Phs-sigV 1L Repeat 1: 3362<br>Phs-sigV 1L Repeat 2: 3510<br>Phs-sigV 1L Repeat 3: 3515	P_{spank}-rsiV: JLB196<br>$P_{hyperspank}$-sigV: JLB219<br>$P_{hyperspank}$-rsi: JLB193

		Phs-sigV 1L Repeat 4: 2947<br>Phs-rsiV 0L Repeat 1: 3264<br>Phs-rsiV 0L Repeat 2: 2319<br>Phs-rsiV 0L Repeat 3: 1399<br>Phs-rsiV 1L Repeat 1: 2734<br>Phs-rsiV 1L Repeat 2: 3317<br>Phs-rsiV 1L Repeat 3: 2148	
S20	Not applicable	N=100	Not applicable
S21	Not applicable	N=100	Not applicable
S22	Not applicable	N=100	Not applicable
S23	Not applicable	N=100 for all subplots	Not applicable
S24	blank 0L 0In: 4<br>blank 1L 0In: 4<br>blank 0L 1In: 4<br>blank 1L 1In: 4<br>WT 0L 0In: 4<br>WT 1L 0In: 4<br>WT 0L 1In: 4<br>WT 1L 1In: 4<br>operon 0L 0In: 4<br>operon 1L 0In: 4<br>operon 0L 1In: 4<br>operon 1L 1In: 4<br>operon 0L 2In: 4<br>operon 1L 2In: 4<br>operon 0L 5In: 4<br>operon 1L 5In: 4<br>operon 0L 10In: 4<br>operon 1L 10In: 4	blank 0L 0I Repeat 1: 4123<br>blank 0L 0I Repeat 2: 4660<br>blank 0L 0I Repeat 3: 2215<br>blank 0L 0I Repeat 4: 3969<br>blank 1L 0I Repeat 1: 4174<br>blank 1L 0I Repeat 2: 3275<br>blank 1L 0I Repeat 3: 4652<br>blank 1L 0I Repeat 4: 1652<br>blank 0L 1I Repeat 1: 4256<br>blank 0L 1I Repeat 2: 4126<br>blank 0L 1I Repeat 3: 3141<br>blank 0L 1I Repeat 4: 3541<br>blank 1L 1I Repeat 1: 2963<br>blank 1L 1I Repeat 2: 5546<br>blank 1L 1I Repeat 3: 3230<br>blank 1L 1I Repeat 4: 2850<br>WT 0L 0I Repeat 1: 2918<br>WT 0L 0I Repeat 2: 3475	blank:JLB43<br>WT:JLB130<br>operon:JLB236

	operon 0L 20In: 4<br>operon 1L 20In: 4<br>operon 0L 50In: 4<br>operon 1L 50In: 4<br>operon 0L 100In: 4<br>operon 1L 100In: 4<br>operon 0L 200In: 3<br>operon 1L 200In: 3<br>operon 0L 500In: 3<br>operon 1L 500In: 3<br>operon 0L 1000In: 4<br>operon 1L 1000In: 4	WT 0L 0I Repeat 3: 3985<br>WT 0L 0I Repeat 4: 2486<br>WT 1L 0I Repeat 1: 2116<br>WT 1L 0I Repeat 2: 3636<br>WT 1L 0I Repeat 3: 5481<br>WT 1L 0I Repeat 4: 2826<br>WT 0L 1I Repeat 1: 4499<br>WT 0L 1I Repeat 2: 3543<br>WT 0L 1I Repeat 3: 5179<br>WT 0L 1I Repeat 4: 2532<br>WT 1L 1I Repeat 1: 4229<br>WT 1L 1I Repeat 2: 3130<br>WT 1L 1I Repeat 3: 4101<br>WT 1L 1I Repeat 4: 2397<br>operon 0L 0I Repeat 1: 5108<br>operon 0L 0I Repeat 2: 3669<br>operon 0L 0I Repeat 3: 4419<br>operon 0L 0I Repeat 4: 1683<br>operon 1L 0I Repeat 1: 5509<br>operon 1L 0I Repeat 2: 4454<br>operon 1L 0I Repeat 3: 4911<br>operon 1L 0I Repeat 4: 2965<br>operon 0L 1I Repeat 1: 4608<br>operon 0L 1I Repeat 2: 5033<br>operon 0L 1I Repeat 3: 4945<br>operon 0L 1I Repeat 4: 3338<br>operon 1L 1I Repeat 1: 7688<br>operon 1L 1I Repeat 2: 6133<br>operon 1L 1I Repeat 3: 6064<br>operon 1L 1I Repeat 4: 3941<br>operon 0L 2I Repeat 1: 6041<br>operon 0L 2I Repeat 2: 4315	

		operon 0L 2I Repeat 3: 3574<br>operon 0L 2I Repeat 4: 434<br>operon 1L 2I Repeat 1: 4557<br>operon 1L 2I Repeat 2: 4851<br>operon 1L 2I Repeat 3: 4235<br>operon 1L 2I Repeat 4: 2615<br>operon 0L 5I Repeat 1: 4921<br>operon 0L 5I Repeat 2: 6273<br>operon 0L 5I Repeat 3: 5774<br>operon 0L 5I Repeat 4: 2824<br>operon 1L 5I Repeat 1: 5825<br>operon 1L 5I Repeat 2: 5089<br>operon 1L 5I Repeat 3: 5178<br>operon 1L 5I Repeat 4: 2912<br>operon 0L 10I Repeat 1: 4359<br>operon 0L 10I Repeat 2: 5366<br>operon 0L 10I Repeat 3: 4622<br>operon 0L 10I Repeat 4: 3813<br>operon 1L 10I Repeat 1: 5343<br>operon 1L 10I Repeat 2: 4772<br>operon 1L 10I Repeat 3: 3304<br>operon 1L 10I Repeat 4: 4035<br>operon 0L 20I Repeat 1: 6554<br>operon 0L 20I Repeat 2: 5535<br>operon 0L 20I Repeat 3: 4553<br>operon 0L 20I Repeat 4: 3381<br>operon 1L 20I Repeat 1: 4322<br>operon 1L 20I Repeat 2: 6707<br>operon 1L 20I Repeat 3: 5988<br>operon 1L 20I Repeat 4: 4072<br>operon 0L 50I Repeat 1: 4989<br>operon 0L 50I Repeat 2: 6820	

		operon 0L 50I Repeat 3: 3615<br>operon 0L 50I Repeat 4: 4361<br>operon 1L 50I Repeat 1: 4995<br>operon 1L 50I Repeat 2: 3282<br>operon 1L 50I Repeat 3: 3921<br>operon 1L 50I Repeat 4: 3417<br>operon 0L 100I Repeat 1: 859<br>operon 0L 100I Repeat 2: 4700<br>operon 0L 100I Repeat 3: 5033<br>operon 0L 100I Repeat 4: 3888<br>operon 1L 100I Repeat 1: 5455<br>operon 1L 100I Repeat 2: 6740<br>operon 1L 100I Repeat 3: 1337<br>operon 1L 100I Repeat 4: 4655<br>operon 0L 200I Repeat 1: 3927<br>operon 0L 200I Repeat 2: 4281<br>operon 0L 200I Repeat 3: 3534<br>operon 1L 200I Repeat 1: 3649<br>operon 1L 200I Repeat 2: 5822<br>operon 1L 200I Repeat 3: 3279<br>operon 0L 500I Repeat 1: 6344<br>operon 0L 500I Repeat 2: 5218<br>operon 0L 500I Repeat 3: 2856<br>operon 1L 500I Repeat 1: 5515<br>operon 1L 500I Repeat 2: 4911<br>operon 1L 500I Repeat 3: 3818<br>operon 0L 1000I Repeat 1: 2616<br>operon 0L 1000I Repeat 2: 5865<br>operon 0L 1000I Repeat 3: 4822<br>operon 0L 1000I Repeat 4: 4976<br>operon 1L 1000I Repeat 1: 4374<br>operon 1L 1000I Repeat 2: 5051	

		operon 1L 1000I Repeat 3: 2244<br>operon 1L 1000I Repeat 4: 4713	
S25	Not applicable	WT: 99<br>2x sigV: 99<br>2x risV: 99<br>2x sigV-rsiV: 99	Not applicable
S26	WT 0 µg/ml n: 3<br>WT 1 µg/ml n: 3<br>2x sigV 0 µg/ml n: 3<br>2x sigV 1 µg/ml n: 3<br>2x rsiV 0 µg/ml n: 3<br>2x rsiV 1 µg/ml n: 3<br>2x sigV-rsiV 0 µg/ml n: 3<br>2x sigV-rsiV 1 µg/ml n: 3	WT 0 Lyso Repeat 1: 483<br>WT 0 Lyso Repeat 2: 673<br>WT 0 Lyso Repeat 3: 439<br>WT 1 Lyso Repeat 1: 499<br>WT 1 Lyso Repeat 2: 624<br>WT 1 Lyso Repeat 3: 365<br>2xsigV 0 Lyso Repeat 1: 495<br>2xsigV 0 Lyso Repeat 2: 353<br>2xsigV 0 Lyso Repeat 3: 280<br>2xsigV 1 Lyso Repeat 1: 454<br>2xsigV 1 Lyso Repeat 2: 443<br>2xsigV 1 Lyso Repeat 3: 400<br>2xrsiV 0 Lyso Repeat 1: 446<br>2xrsiV 0 Lyso Repeat 2: 353<br>2xrsiV 0 Lyso Repeat 3: 327<br>2xrsiV 1 Lyso Repeat 1: 510<br>2xrsiV 1 Lyso Repeat 2: 674<br>2xrsiV 1 Lyso Repeat 3: 502<br>2xsigVrsiV 0 Lyso Repeat 1: 402<br>2xsigVrsiV 0 Lyso Repeat 2: 550<br>2xsigVrsiV 0 Lyso Repeat 3: 431<br>2xsigVrsiV 1 Lyso Repeat 1: 434<br>2xsigVrsiV 1 Lyso Repeat 2: 475<br>2xsigVrsiV 1 Lyso Repeat 3: 342	WT: JLB130<br>2x sigV: JLB210<br>2x risV: JLB212<br>2x sigV-rsiV: JLB211
S27	Not applicable	100 for all subplots	Not applicable

S28	1<br>The other repeat is in Figure 6 of the main text	6h no stress: 48<br>12h no stress: 50<br>Control: 50	JLB221
S29	1	Memory 6h: 20<br>Memory 12h: 26	JLB130
S30	A) n=2<br>B) n=3	A)<br>0 µg/ml Repeat 1: 48<br>0 µg/ml Repeat 2: 34<br>0.5 µg/ml Repeat 1: 40<br>0.5 µg/ml Repeat 2: 43<br>1 µg/ml Repeat 1: 47<br>1 µg/ml Repeat 2: 38<br>2 µg/ml Repeat 1: 47<br>2 µg/ml Repeat 2: 44<br>4 µg/ml Repeat 1: 48<br>4 µg/ml Repeat 2: 43<br><br>B) not applicable	A) WT: JLB130<br>B) WT:JLB130<br>isigV: JLB219<br>ioatA: JLB218
S31	3	WT 1 Lyso Repeat 1: 841<br>WT 1 Lyso Repeat 2: 1141<br>WT 1 Lyso Repeat 3: 961<br>ΔepsH 1 Lyso Repeat 1: 1219<br>ΔepsH 1 Lyso Repeat 2: 1617<br>ΔepsH 1 Lyso Repeat 3: 1104	WT: JLB130<br>ΔepsH: JLB221
S32	A-E) 2	A-E)<br>WT Repeat 1: 50<br>ΔepsH Repeat 1: 50	WT: JLB130<br>ΔepsH: JLB221

		WT Repeat 2: 53<br>ΔepsH Repeat 2: 56	
S33	Not applicable	N=100	Not applicable
