# Supplementary material for: Tunable phenotypic variability through an autoregulatory alternative sigma factor circuit
Source: Mol Syst Biol. 2021 Jul 19;17(7):e9832. doi: 10.15252/msb.20209832 (PMC8287880; doi:10.15252/msb.20209832)
Supplement: Supplementary file 2 — Expanded View Figures PDF [file MSB-17-e9832-s001.pdf]

Expanded View Figures

Figure EV1. With increasing stress levels, the heterogeneity in σ^V activation times is reduced.

In each subpanel, a line corresponds to a single-cell trace of one mother cell in the mother machine ($N = \sim 50$). The stress was added after 240 min (dashed line). Once P_{sigV}-YFP passed its half maximum the traces end.

A, B Single-cell traces in response to 0.5 μg/ml lysozyme.
C, D Single-cell traces in response to 1 μg/ml lysozyme.
E, F Single-cell traces in response to 2 μg/ml lysozyme.
G, H Single-cell traces in response to 4 μg/ml lysozyme.

Data information: The data are the same as in *n* For more information on the number of repeats, please see Appendix Table S4.

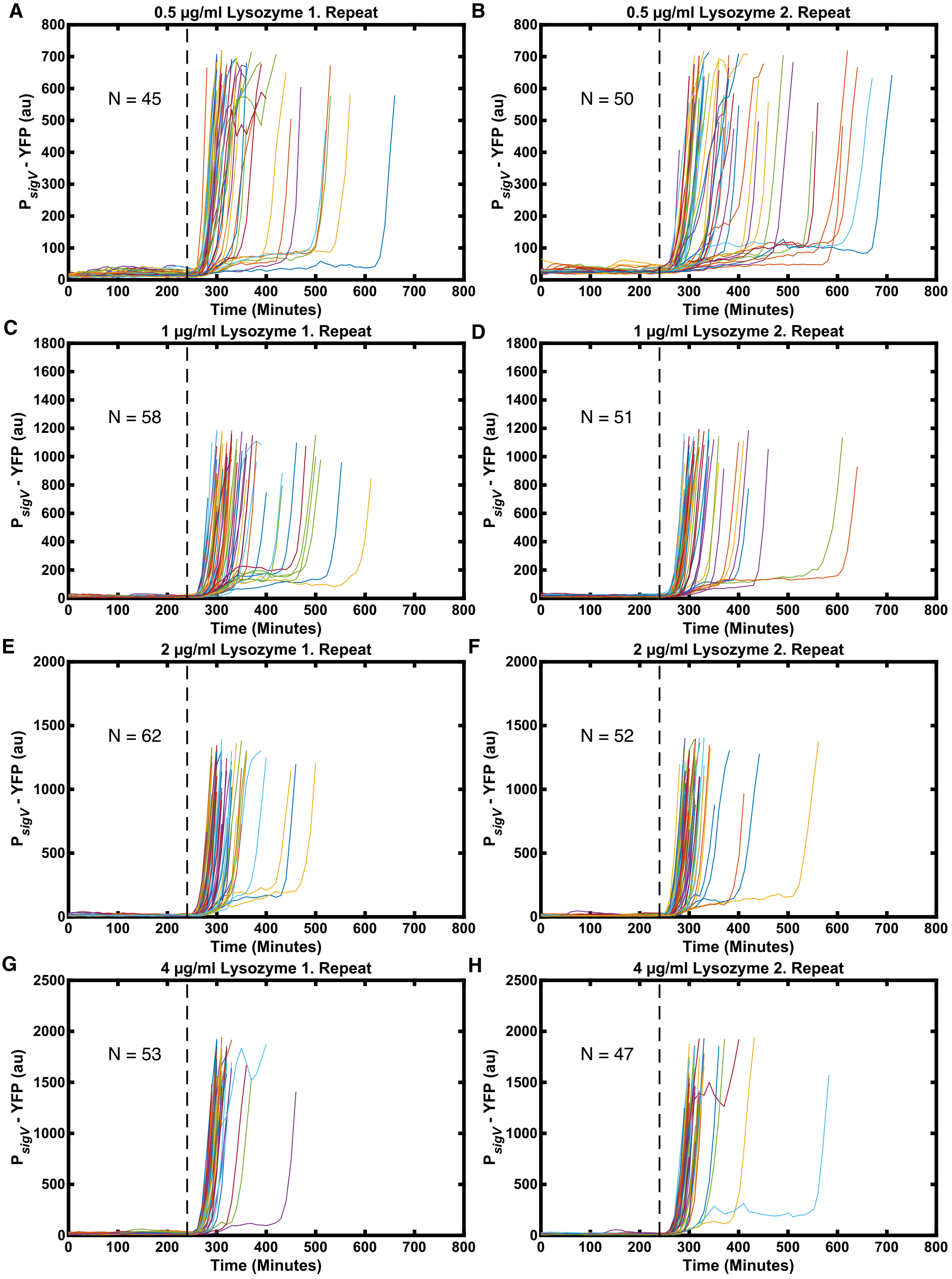

Figure EV1.

 © 2021 The Authors

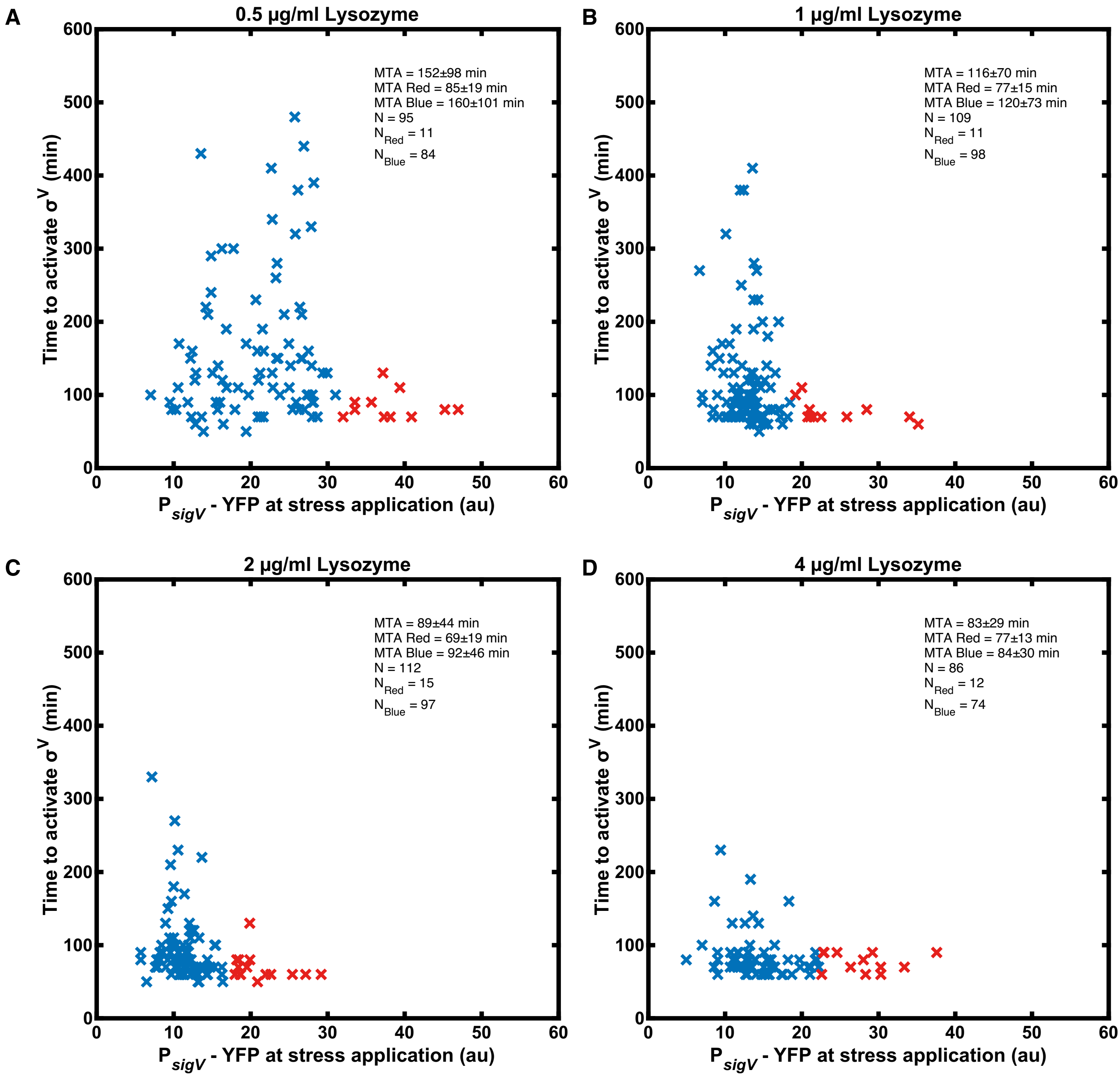

Figure EV2. Cells with high P_{sigV}-YFP before stress activate σ^V rapidly.

Figure plots P_{sigV}-YFP levels at the time of stress addition, against the time to activation. Cells with a YFP fluorescence larger than the mean + s.d. (of the pre-stress YFP distribution) are marked with red crosses. All other cells are marked with blue crosses. In the inset MTA stands for mean time to activate.

A Activation time for 0.5 µg/ml lysozyme.
B Activation time for 1 µg/ml lysozyme.
C Activation time for 2 µg/ml lysozyme.
D Activation time for 4 µg/ml lysozyme.

Data information: All shown data in the plots are from two biological repeats. For more information on the number of repeats, please see Appendix Table S4.

© 2021 The Authors

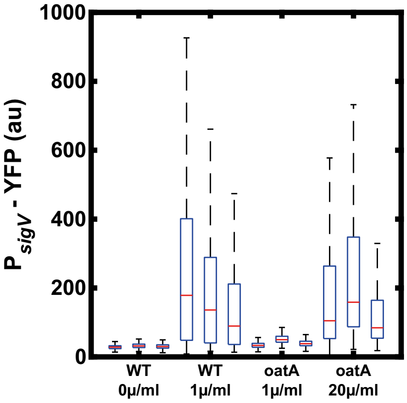

Figure EV3. Overexpressing *oatA* shuts off σ^V activation.

Increasing stress levels from 1 to 20 µg/ml recaptures the WT σ^V activation dynamics when *oatA* is overexpressed. $n = 3$ biological repeats for all data shown. On each box, the central mark indicates the median, and the bottom and top edges of the box indicate the 25th and 75th percentiles, respectively. The lower and higher whiskers of boxplot are extended to the first quartile minus 1.5 * interquartile range and the third quartile plus 1.5 * interquartile range, respectively.

Data information: For more information on the number of repeats, please see Appendix Table S4.

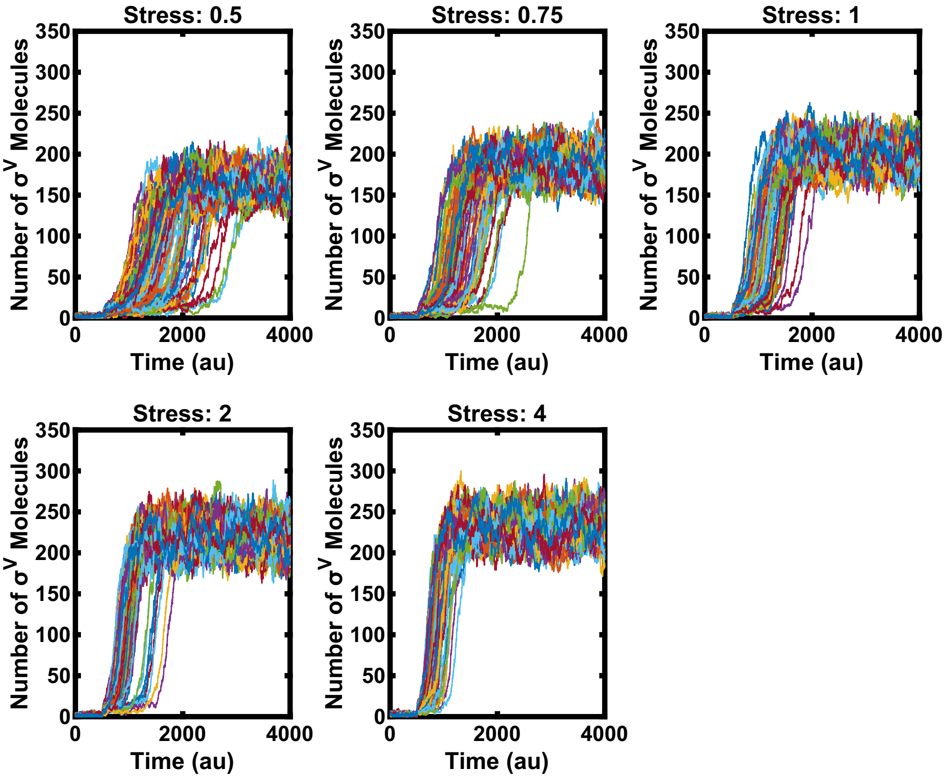

Figure EV4. With increasing stress levels, the heterogeneity in activation is reduced in the mathematical model.

Each line corresponds to one simulated cell. The stress was added (parameter *L* changed from 0 to 1) at time point 500 ($N = 100$).

Data information: For more information on the number of repeats, please see Appendix Table S4.

© 2021 The Authors

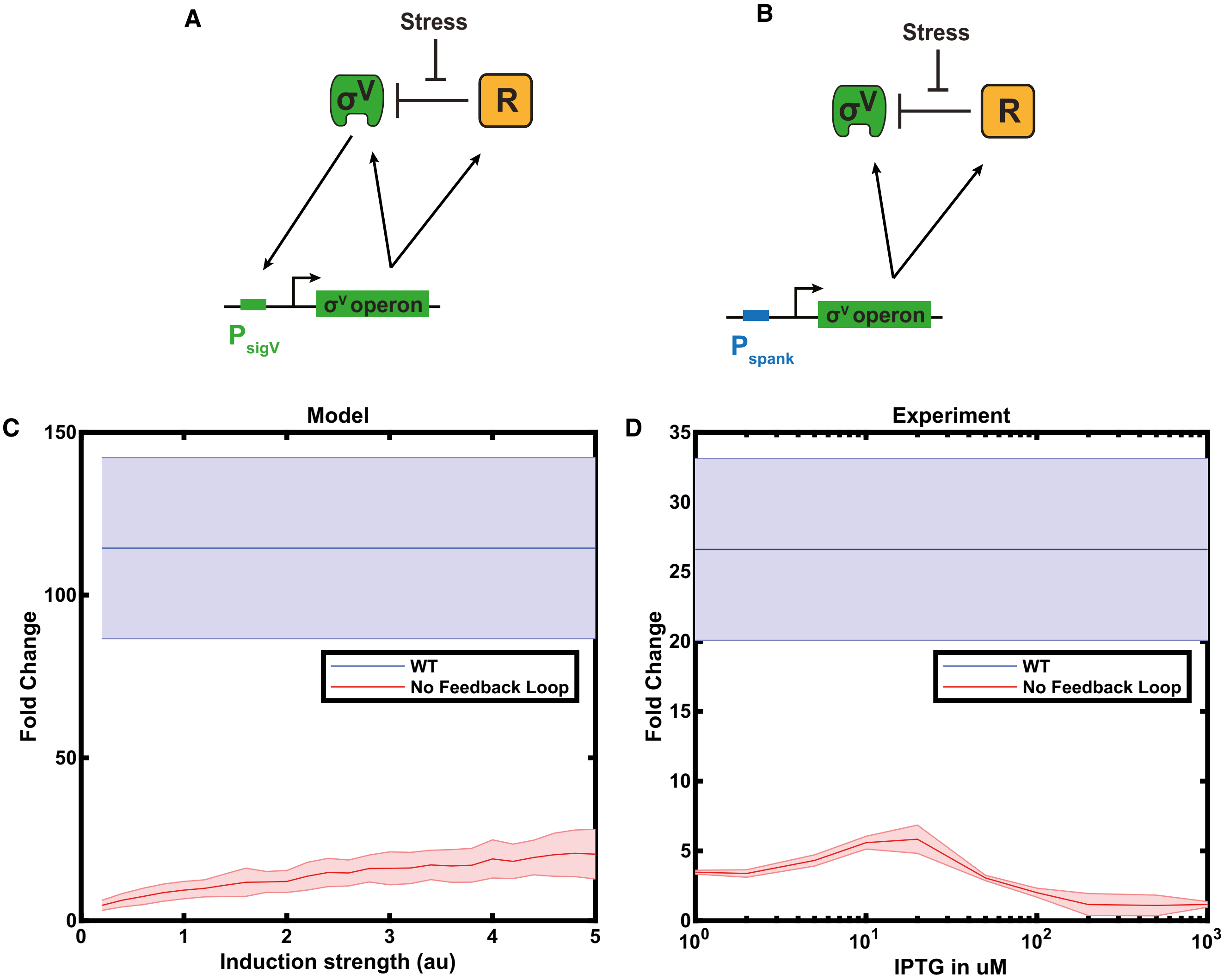

Figure EV5. The *sigV* feedback loop increases the dynamic range of the circuit.

A Sketch of WT *sigV* circuit. Where R stands of RsiV.

B Sketch of rewired circuit with deleted positive feedback loop. Where R stands for RsiV and P_{spank} for the IPTG inducible promoter spank.

C The fold change in *sigV* expression pre/post 1 μg/ml lysozyme stress was calculated from model simulations of the WT system (blue line) and a feedback-broken system where the levels of the *sigV* operon are inducible (red line). The shaded areas represent one standard deviation of mean. $N = 100$ simulation.

D The experimental fold change for the WT, and the feedback-broken strain for IPTG values between 0 to 1,000 μM. The blue line is the WT fold change, and the red line is the fold change of Δ*sigVrsiVoatAyrhK* P_{spank}-*sigVrsiVoatAyrhK*. The shaded areas represent one standard deviation of mean. $n = 3$ biological repeats for all data shown.

Data information: For more information on the number of repeats, please see Appendix Table S4.

© 2021 The Authors
