# Supplementary material for: Tunable phenotypic variability through an autoregulatory alternative sigma factor circuit
Source: Mol Syst Biol. 2021 Jul 19;17(7):e9832. doi: 10.15252/msb.20209832 (PMC8287880; doi:10.15252/msb.20209832)
Supplement: Supplementary file 3 — Movie EV1 [file MSB-17-e9832-s003.zip › MSB-20-9832-MovieEV1.rtf]

Movie EV1. óV is activated heterogeneously in response to lysozyme stress. 
JLB130 is grown in the mother machine microfluidic device. The constitutively expressed RFP (magenta) and PsigV-YFP (green) ranges were chosen for display. The imaging interval is 10 minutes.
