# Supplementary material for: Tunable phenotypic variability through an autoregulatory alternative sigma factor circuit
Source: Mol Syst Biol. 2021 Jul 19;17(7):e9832. doi: 10.15252/msb.20209832 (PMC8287880; doi:10.15252/msb.20209832)
Supplement: Supplementary file 4 — Movie EV2 [file MSB-17-e9832-s004.zip › MSB-20-9832-MovieEV2.rtf]

Movie EV2. After a 6 h break in stress, óV activation is homogenous. 
JLB221 is grown in the mother machine microfluidic device. The cells experience lysozyme stress from the beginning of the movie. At time point 0 min stress is removed and then readded 6 h later.  The constitutively expressed RFP (magenta) and PsigV-YFP (green) ranges were chosen for display. The imaging interval is 10 minutes.
